# Conceptualising, operationalising, and measuring trust in participatory health research networks: a scoping review

**DOI:** 10.1186/s13643-022-01910-x

**Published:** 2022-03-06

**Authors:** Meghan Gilfoyle, Anne MacFarlane, Jon Salsberg

**Affiliations:** Public & Patient Involvement Research Unit, School of Medicine and Health Research Institute (HRI), University of Limerick, Limerick, Ireland V94 T9PX

**Keywords:** Community-based participatory research, Trust, Social networking, Patient participation, Community participation, Review

## Abstract

**Background:**

There are many described benefits of community-based participatory research (CBPR), such as increased relevance of research for those who must act on its findings. This has prompted researchers to better understand how CBPR functions to achieve these benefits through building sustainable research partnerships. Several studies have identified “trust” as a key mechanism to achieve sustainable partnerships, which themselves constitute social networks. Although existing literature discusses *trust and CBPR*, or *trust and social networks*, preliminary searches reveal that none link all three concepts of *trust*, *CBPR*, and *social networks.* Thus, we present our scoping review to systematically review and synthesize the literature exploring how trust is conceptualised, operationalised, and measured in CBPR and social networks.

**Methods:**

This review follows the guidance and framework of Peters et al. which is underpinned by the widely used framework of Levac and colleagues. Levac and colleagues provided enhancements to the methodological framework of Arksey and O’Malley. We explored several electronic databases including Scopus, Medline, PubMed, Web of Science, CINAHL, Cochrane Library, Google Scholar, and PsychINFO. A search strategy was identified and agreed upon by the team in conjunction with a research librarian. Two independent reviewers screened articles by title and abstract, then by full-text based on pre-determined exclusion/inclusion criteria. A third reviewer arbitrated discrepancies regarding inclusions/exclusions. A thematic analysis was then conducted to identify relevant themes and sub-themes.

**Results:**

Based on the 26 extracted references, several key themes and sub-themes were identified which highlighted the complexity and multidimensionality of trust as a concept. Our analysis revealed an additional emergent category that highlighted another important dimension of trust—outcomes pertaining to trust. Further, variation within how the studies conceptualised, operationalised, and measured trust was illuminated. Finally, the multidimensionality of trust provided important insight into how trust operates as a context, mechanism, and outcome.

**Conclusions:**

Findings provide support for future research to incorporate trust as a lens to explore the social-relational aspects of partnerships and the scope to develop interventions to support trust in partnerships.

**Supplementary Information:**

The online version contains supplementary material available at 10.1186/s13643-022-01910-x.

## Background

Participatory research (PR) is defined as the “systematic inquiry, with the collaboration of those affected by the issue being studied, for the purposes of education and taking action or effecting change” [1]. In recent decades, participatory research (PR) has been gaining recognition throughout research communities as an approach that serves to bridge the gap between research and practice [2, 3]. Specifically, PR helps to maximise the relevancy of research and usability of its products, while simultaneously building capacity and addressing issues of social justice and self-determination among end-user communities [2, 3]. Currently, an overarching theory of PR does not exist, underscoring the need for greater knowledge of the key concepts and mechanisms of participatory research.

This is challenging as there are many different labels that exist that fall within the realm of participatory research, (e.g., public and patient involvement, participatory health research, participatory action research), all striving to bridge this gap between knowledge and practice by promoting inclusivity, while ensuring all partners who the research serves to benefit are actively engaged in the research process [3].

Despite this challenge, there have been important advancements towards theory development in PR. One such advancement comes from one of the more widely recognized bodies of literature within PR falling under the heading of community-based participatory research (CBPR), with core philosophy and values grounded in social and environmental justice and self-determination to address inequities, particularly in regards to health [3]. Similarly, the W.K. Kellogg Foundation’s Community Health Scholars Program [4] defines CBPR as:

A collaborative approach to research that equitably involves all partners in the research process and recognizes the unique strengths that each brings. CBPR begins with a research topic of importance to the community with the aim of combining knowledge and action for social change to improve community health and eliminate health disparities [4].

For this scoping review, we will use the term CBPR as an all-encompassing term, which like PR, will incorporate a broad range of terms (e.g., public and patient involvement, participatory health research, participatory action research), that embrace shared core philosophies and values. CBPR was chosen as the term of choice for this review given its wide recognition across the literature, and its associated conceptual model [2, 3, 5].

Specifically, a CBPR conceptual model was developed [5] and adapted [2] which provides a concrete framework for understanding how the CBPR process is influenced by contextual and process-related aspects that can affect the ability to achieve both intermediate impacts (e.g. stronger partnerships) and long-term outcomes (e.g. improved health, community transformation, and health equity) [6]. The CBPR conceptual model was deemed appropriate for addressing key gaps in the literature because of its comprehensive nature and its focus on the relationship between context, process dynamics, and research outcomes [7]. These gaps include theoretically and empirically explaining “how contexts, partnership practices, and research/intervention engagement factors contribute to broad-based CBPR and health outcomes” [7]. Oetzel et al. [7] empirically tested variables of the CBPR model, with the aim “to better understand the mechanisms for impact on achieving” intermediate and long-term health outcomes, such as community transformation. Findings from this study found that the model was suitable for explaining important *relational* (e.g. interactive) and *structural* (e.g. team composition and nature) processes [2] and pathways for impact on intermediate and long-term outcomes [7].

With an emphasis on the relational aspect of the CBPR model, a systematic review by Jagosh et al. [8] identified partnership synergy as a universal feature of the collaborative process necessary for building and sustaining partnerships that create resilience, sustain health-related goals, and extend program infrastructure, while creating new and unexpected ideas and outcomes. Literature from the community perspective includes various accounts of community problems of engagement and trust. Jagosh et al. [9], for example, identify instances where contextual factors such as history of oppression or research abuse have triggered mistrust in the community, impacting positive outcomes, such as partnership synergy. Jagosh et al. [9] further explored what supports partnership synergy in successful long-term CBPR partnerships. The building and maintenance of *trust* was identified as a key mechanism in this process. However, Jagosh et al. [9] treated trust as a “black box” concept without unpacking its internal dimensions and processes. This limits understanding/progress because if there is no clear conceptualisation of trust then it is challenging to operationalise or measure it in real-world partnerships.

Therefore, it is valuable to explore how trust is conceptualised, operationalised, and measured in CBPR partnerships. To do this, a methodology must be adopted that supports the analysis of trust in CBPR partnerships.

It is necessary to describe and measure trust among and between research partners within CBPR. Conceptually, a social network can be seen as a set of connections between individuals or organisations. This is similar to a partnership, where individuals or organisations are connected around a common purpose [10]. Social network analysis (SNA) is a methodology for describing and measuring contextual and relational dynamics among and between social actors [11]. SNA provides tools for investigating the development and maintenance of trust and trustworthiness and their effects on partnership functioning within social networks [12]. The potential value here is, for example, as a CBPR project unfolds, the ability to measure trust can allow for the design of structural interventions (e.g. adding or removing planned working meetings) to improve trust and partnership function by supporting context or social structures within the partnership [8, 9].

**Fig. 1:**
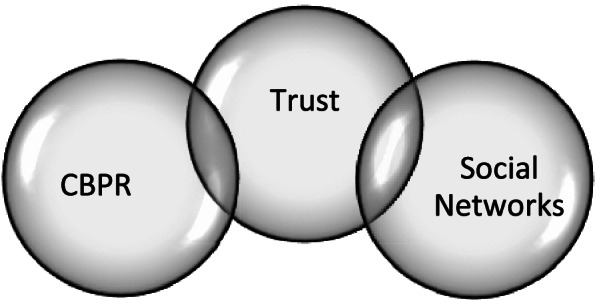
Trust, CBPR, and social networks as a conceptual triad

Social networks have been used to explore trust in diverse fields, such as in health [13] or education [14]. They have also been used to explore dynamics within CBPR [15, 16]. However, social networks have not been used to explore the dynamics of trust *within* CBPR. Therefore, CBPR, social networks, and trust (Fig. 1) constitute a conceptual triad that may allow us to better understand how partnership function leads to better research outcomes.

### Purpose of conducting the scoping review

Although existing literature discuss *trust and CBPR* [17], or *trust and social networks* [18], preliminary searches revealed that none of the literature explores all three concepts of *trust*, *CBPR*, *and social networks*. Furthermore, preliminary searches revealed a lack of consensus regarding how trust is conceptualised, operationalised, and measured. With this in mind, the objectives of this scoping review were to:

1. Identify the literature on trust in CBPR and social networks
2. Clarify how trust is conceptualised, operationalised, and measured in CBPR and social networks
3. Identify where these dimensions of trust may intersect across both CBPR and social networks

Table 1 presents the definitions and boundaries that guided how we conceptualised, operationalised, and measured trust in our scoping review.

**Table 1:** Boundaries and definitions for the conceptualising, operationalising, and measurement of trust in our scoping review

Dimension of our research question	The definition we attached to this dimension of our research question	The boundary for data extraction to inform understanding of the research question dimension
Conceptualisation	Assigning meaning to something	Definition of trust
Operationalisation	Selecting observable phenomena to represent abstract concepts How will we go about empirically testing the concept?	Dimensions and indicators of trust What are the operationalisation issues with the concept? • Based on our indicators, what questions were asked to represent trust, what observations were made, what specific attributes will exist for the measure used?
Measurement	Process of observing and recording the observations, or assigning numbers to a phenomenon	Level of measurement such as nominal, ordinal, interval or ratio and type of measures such as survey, scaling, qualitative, unobtrusive used for trust

### Review question

To clearly identify our research question guiding the scope of the review, we iteratively searched and revised our search terms to capture the most appropriate body of literature. When forming the research question, we identified our main concept of trust and two principal contextual settings for which the concept was explored: CBPR and social networks. The broad nature of these concepts was important in capturing a breadth of literature [19]. This is followed by addressing our target population, being all human studies. Finally, our outcome of interest was to use the literature to see how social network research and CBPR intersect in their conceptualisation, operationalisation, and methods of measurement for trust. This led to the formulation of our research question:

How does the literature conceptualise, operationalise, and measure trust within the context of community-based participatory health research and social networks?

### Eligibility criteria

Deliberation among two additional members of the research team regarding exclusion and inclusion criteria at the outset of the scoping review process occurred. Table 2 provides an overview of the eligibility criteria for this scoping review.

**Table 2:** Eligibility criteria

Criterion	Inclusion	Exclusion	Justification
Population and Sample	Humans	Any study population other than humans, i.e. animal studies	Referring to CBPR partnerships between humans
Language	Written in English	Any other language that is not written in English	Reviewers only speak English
Time Period	1995–2020	Outside this time period	• Still able to capture a wide breadth of literature within the time when CBPR research became more prominent and defined by the pioneers in the field • Our definition of CBPR is consistent with that defined by Lawrence W. Green and colleagues [1] in the 1995 text “Study of participatory research in health promotion: review and recommendations for development of participatory research in health promotion in Canada”
Study Focus	1) Articles that discuss participatory health research and trust OR 2) Articles that discuss social networks and trust	1) Must be participatory health research, not other forms of participatory research outside of the health context OR 2) Social networks across a variety of disciplines, excluding those with a sole focus on online social networks using platforms such as Facebook, Instagram, and Twitter, with no reference to conceptualising (operationalising or measuring) trust in a relational context 3)Trust is explored in a natural setting and not a laboratory or experimental setting (i.e. a game theory setting) 4)Exclude literature that explores trust in social networks, where trust is the independent variable	1) One key reason participatory research was developed, historically, was to address social inequities [2, 3] • Ensuring continuity in conceptualisations from the literature to inform the formation of a conceptual framework for participatory health research 2) In our study context, and the context of CBPR more generally, interactions and partnership building are usually about interpersonal face-to-face contact and communication, which is not adequately reflected in social media networks, such as Facebook and Twitter • Online social network platforms (like those above) are looking at social phenomena unrelated to the type of interactions we are interested in uncovering (such as, creating online trust communities, where people share thoughts and opinions with others they may not know, or have had a face-to-face interaction with) [20] 3) Artificial settings may not adequately reflect our study context, for similar reasons to that of online social networks 4)In our study context, we are interested in discovering variables that altered the level of trust, and thus discovering what can promote/discourage trust in a social network
Type of article	Peer reviewed journal articles or reviews and grey literature. Specifically, grey literature will include theses/dissertations, reports, conference proceedings, editorials, and chapters in a textbook	Any other literature that is not listed in the inclusion criteria, such as websites	• Scoping reviews aim to capture more than peer reviewed and published literature to expansively explore a broad research question • Preliminary searches of grey literature generally revealed those listed in our inclusion criteria • Acknowledging feasibility and time constraints, we felt the literature criteria listed would be sufficient in capturing the necessary literature to inform our review and ultimately, a conceptual framework
Geographic Location	Any location—an international context	None	Participatory research has applications globally

## Methods

This scoping review follows the guidance and framework of Peters et al. [21], which is underpinned by the widely used framework of Levac and colleagues [22]. Levac and colleagues provided enhancements to the methodological framework of Arksey and O’Malley [23]. A published protocol is available for this scoping review [24].

### Search strategy

As initially discussed by Arksey and O’Malley [23], it was important for us to clearly define the terminology we used when conducting the literature search as it ensured the syntax used appropriately captured the literature that best reflected our research question and objectives. Identifying our search strategy was an iterative process that, as proposed by Levac et al. [22], was a team approach. In alignment with the guidelines from Peters et al. [21], a three-step process was used to identify the search strategy.

First, we conducted a preliminary search in CINAHL and Medline searching article titles, abstracts, keywords, and subject headings to guide the development of our search strategy. Secondly, we included the identified keywords and subject headings from the search strategy across all databases being used. Finally, we looked at the reference lists from articles selected for the review. A faculty librarian also provided suggestions and verifications regarding the appropriate syntax and the adaptation of search strategies across databases.

Our final search strategy involved a combination of the three overarching concepts, including concept 1: community-based participatory health research, concept 2: trust, and concept 3: social networks. Literature needed to include either CBPR (concept one) and trust (concept two) in the title or abstract OR social networks (concept three) and trust (concept two) in the title or abstract:

[((“action research OR community-based participatory research OR public and patient involvement) OR (participatory health research” AND “trust or trusting or trustworthiness or trustworthy”)), OR ((“social network or social networks”) AND (“trust or trusting or trustworthiness or trustworthy”))]

This strategy was used across all databases, with slight refinements to match each of the database requirements. The above search string was used in CINAHL.

Recognizing that comprehensiveness is a key strength of a scoping review, we wanted to ensure data sources were heterogeneous, while not compromising feasibility. With that in mind, we explored several electronic databases including Scopus, Medline, PubMed, Web of Science, CINAHL, Cochrane Library, Google Scholar, and PsychINFO. We also included grey literature such as theses/dissertations and reports. We did not require a separate database to capture additional grey literature, as we felt it was adequately captured in Google Scholar and CINAHL. A complete search strategy from one of the major databases used (CINAHL) is outlined in Additional File 1.

### Source of evidence screening and selection

The resulting literature from each of the aforementioned databases was uploaded to the systematic review software “DistillerSR” (https://www.evidencepartners.com/products/distillersr-systematic-review-software/). Once duplicates were removed, two independent reviewers screened the articles by title and abstract and then at full-text based on the pre-determined eligibility criteria, outlined in Table 2.

Noticing the vast amounts of articles to be screened at full-text, more of which involved trust and social networks, we decided that the literature addressing trust in social networks must have included two out of three of our research question components: how trust conceptualised, operationalised, and measured in social networks to be eligible for inclusion. However, for literature addressing PHR and trust, only one of these components needed to be addressed for inclusion. This was considered important to balance the representation of literature from both SN and CBPR in our review.

As anticipated, as the review process progressed, along with our sense of the literature that existed in these areas (trust in CBPR and/or trust in social networks), further changes to the existing eligibility criteria occurred to refine our scope. First, we were interested in exploring trust in social networks as it occurs naturally in relationships. Therefore, studies that included artificial settings, such as experiments that explored “game theory” as a method of exploring trust, were excluded. Second, given the abundance of literature deemed eligible for trust and social networks based on our eligibility criteria, we added additional criteria to further refine our selection for literature about these concepts. Specifically, we discovered that the more suitable literature involved studies that explored trust as a dependent variable as we wanted to see the effect that other variables had on trust and thus how the strength or level of trust was altered when the independent variable was manipulated. Thus, if trust was the independent variable in the literature being reviewed, it was deemed ineligible and excluded. Finally, after the full-text review was completed, we still found we had an over-abundance of items that matched our inclusion criteria. This created the opportunity to be more selective and only retain items that more closely addressed our research question. Thus, we created an additional full-text review stage that only included literature that addressed all three of the components from our research question (how trust was conceptualised, operationalised, and measured) for trust in social networks and two of the three components for trust in PHR.

**Fig. 2:**
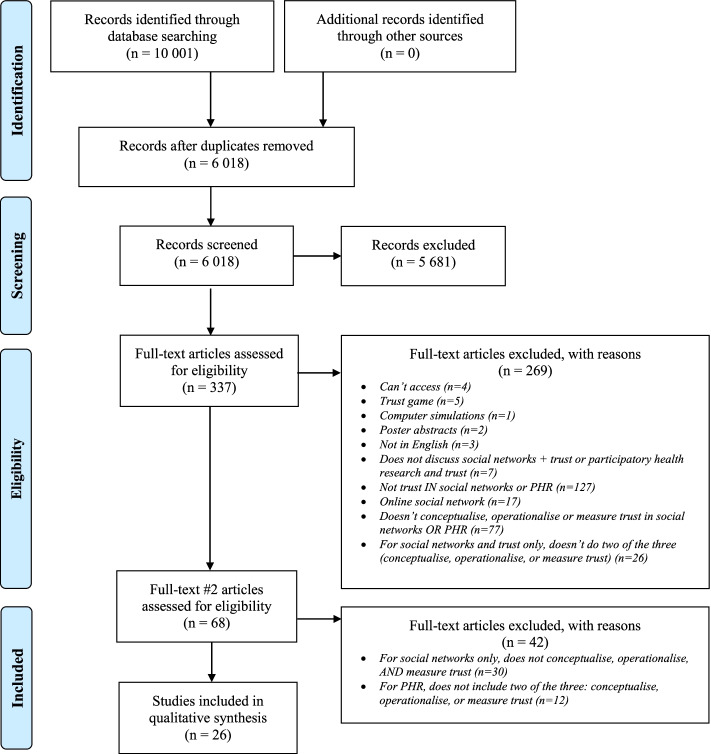
PRISMA 2009 flow diagram. From: Moher D, Liberati A, Tetzlaff J, Altman DG, The PRISMA Group (2009). Preferred Reporting Items for Systematic Reviews and Meta-Analyses: The PRISMA Statement. PLoS Med 6(7): e1000097. 10.1371/journal.pmed1000097. For more information, visit www.prisma-statement.org

The pair of reviewers met at multiple stages throughout the reviewing process to discuss any discrepancies and changes in eligibility criteria that emerged. Any existing discrepancies regarding which articles to include or exclude and/or why were deemed a “conflict” and subsequently sent to a third independent reviewer who made the final decision. See Fig. 2 below for the PRISMA [25] flow diagram which includes the finalised numbers of what was included and excluded at each stage of the review process.

### Data extraction

To ensure that the most suitable information was extracted, a tabular chart organised in Excel, following guidelines from Peters et al. [21], was incorporated and adapted to include an additional column pertaining to associated questions guiding the charting elements, as illustrated in the protocol by Nittas et al. [26]. Furthermore, additional rows were added that discussed in which context the article was addressing trust, as well as how trust was conceptualised, operationalised, and measured in these contexts. This additional information was important to note for the subsequent stage of the review process; collating, summarizing, and reporting the results (identifying themes). One reviewer completed the data charting process, which was an iterative process as new data was presented in the examination stages, leading to continual charting updates.

### Analysis and presentation of results

As suggested by Peters et al. [21], a narrative summary was included to complement the tabular results and discuss how the findings relate to the research question and objectives. In addition to this descriptive narrative summary, we also conducted a thematic analysis of the literature using qualitative description [27]. The thematic analysis followed the guidance of Braun and Clarke [28, 29]. We understood the importance of not pre-empting the findings of the scoping review therefore employed strategies from Braun and Clarke [28, 29] such as “A 15-point checklist of criteria for good thematic analysis” [28, 29] to ensure rigour in collating and summarizing the results. NVIVO software was used to analyse extracted data into themes and subthemes which are further explored in the results section of this review. Findings were organised into thematic categories including methodological design and key findings, but also by categories that specifically highlighted the theoretical and operational linkages such as context, conceptual and operational features, and measurements used.

#### Consultation with knowledge users

As initially suggested by Levac et al. [22], and later underscored by Peters et al. [21], we recognised that consultation with knowledge users adds to the methodological rigour of a study and should be included as a non-optional stage in developing a scoping review. This review is part of a larger participatory health research project involving 11 collaborating stakeholders that are representatives from community and patient organisations, as well as academic and health services entities that comprise the public and patient involvement capacity building team at the University of Limerick (known as “PPI-Ignite@UL”). There was consultation with them regarding whether or not to conduct the review and if the topic seemed novel and applicable within the scope of the larger study. Indeed, results from this scoping review will be returned to these stakeholders, where feedback will be provided, which will then feed into a larger study based on results from this scoping review.

## Results

### Search results

The search strategy used generated a total of 10 001 references. Once these were screened for duplicates, a total of 6 018 references were eligible to be screened by title and abstract. When screening by title and abstract, 5 681 references were removed as they did not meet our eligibility criteria described in Table 2. This left 337 articles to be screened at full text. A total of 269 articles were excluded after being reviewed at full text. The predominant reason for exclusion was that trust was not being discussed in social networks or not explored within the CBPR partnerships (i.e. two concepts explored in parallel and not together) (*n* = 127, 47%). The second highest reason for exclusion was that the article did not conceptualise, operationalise or measure trust in social networks or CBPR (*n* = 77, 29%). The remaining reasons for exclusion can be found in Fig. 2 PRISMA 2009 Flow Diagram and the PRISMA-ScR checklist can be found in Additional File 2. As we still had 68 articles remaining, we further refined our screening criteria to achieve a smaller sample for more in-depth analysis and added an additional full-text review stage. Specifically, as explained earlier in the Methods section, given the overabundance of retained items with a social network and trust focus at this stage, we had the opportunity to further refine our inclusion criteria. Thus, for social network and trust articles, if trust was not conceptualised, operationalised, *and* measured it was excluded (*n* = 30, 71%). Meanwhile, for CBPR-related articles, as there was not an overabundance, if *two* of three (conceptualisation, operationalisation, or measurement) of trust was not present, it was excluded (*n* = 12, 29%). After this final review stage was completed, 26 items remained and were included for data extraction and qualitative synthesis.

### Inclusion of sources of evidence

**Objective #1:** Identify the literature on trust in CBPR and social networks

From the included literature (*n* = 26) [30–54], 20 references [30–42, 44–46, 50–53] were peer-reviewed journal articles, four references [47–49, 55] were dissertations, one reference [43] was a systematic review, and one reference [54] was a book chapter. The included references were published between 2005 and 2019 [30–54]. The majority of references explored trust in social networks (*n* = 17) [30–35, 38–42, 49–54], while seven references explored trust in CBPR [43–48, 55], and two explored both trust in social networks and CBPR [36, 37]. Further individual study details and characteristics can be found in Additional File 3.

### Review of findings

**Objectives #2 and #3**—How is trust conceptualised, operationalised, and measured in CBPR and social networks? Identify where these dimensions of trust may intersect across both CBPR and social networks.

**Fig. 3:**
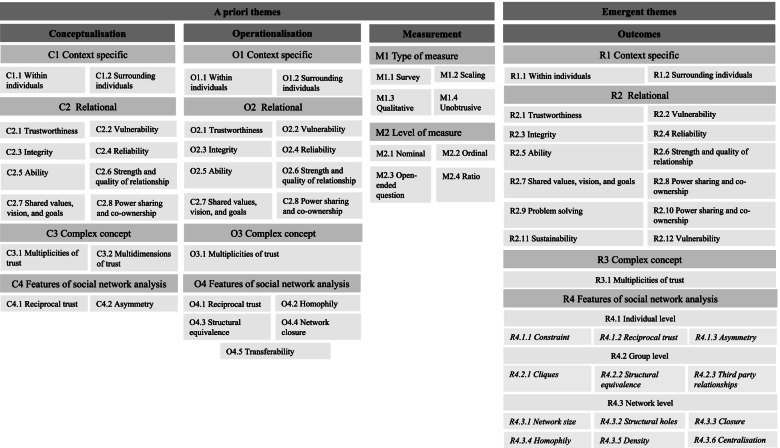
Themes and sub-themes (a priori; emergent)

Findings from the thematic analysis exploring how trust is conceptualised, operationalised, and measured for each extracted reference can be found in Additional File 3. For each reference, there are columns illustrating apriori themes—how trust was conceptualised, operationalised, measured. The outcomes pertaining to trust was an emergent theme. Figure 3 shows the identified parent themes and sub-themes. For example, for the conceptualisation of trust, four parent themes were revealed: C1 “context-specific”, C2 “relational”, C3 “complex concept”, and C4 “features of social network analysis.” Subsequently, sub-themes attached to each parent-theme were identified. This format of parent-themes and sub-themes is similar for operationalisation, measurement, and outcomes pertaining to trust.

### Trust: Conceptualised

When observing the themes and sub-themes presented in Fig. 3, we begin to see the complexities of trust, by noting the many features involved when defining trust as a concept (for detailed descriptions of all themes and sub-themes for conceptualisation, please refer to Table 3).

**Table 3:** Findings for conceptualisation of trust

Conceptualisation: how does the study define trust?			
Themes and sub-themes		Description	References
Theme C1	Context specific	This theme describes trust as a concept that is affected by the given context. The context varies depending on the traits that exist within an individual as a kind of precondition to trust, as well as the context surrounding an individual, such as individuals in their network.	
*ST C1.1*	*Within individuals*	This sub-theme describes how individuals within a context, and thus the traits that exist within the individual, define trust. Specifically, trust can be dependent on the individual’s personality and experiences which can impact their disposition to trust. For example, trust can be influenced by their past experiences with trust (or mistrust) in others (i.e. groups, individuals, and organisations).	“First, initial trust depends on personality; people simply differ in their general disposition to trust/distrust.” [32] “If no other situational information is available, one will rely on one’s general belief that nonspecific individuals can be trusted” [32]
*ST C1.2*	*Surrounding individuals*	This sub-theme describes how the context surrounding an individual can influence trust. For instance, the norms, values, setting, institutional barriers, and level of support from others surrounding the individual in a given environment can influence trust.	“Trust (40 out of 95 items) is defined as the degree to which core group members from the health organization(s) and academic institution(s) feel that the partnership provides a supportive environment”,] [43] “According to members of community-academic partnerships trust is having an emotionally safe and respectful environment” [55] “trust must be understood from the perspective of all parties and within its context” [44]
Theme C2	Relational	This theme focuses on the notion that trust plays an important role in relationships and thus is generally referred to as an interpersonal concept. Specifically, trust is a fluid concept from a relational perspective, involving a variety of features that require and depend on another individual (ie., trustor to trustee).	
*ST C2.1*	*Trustworthiness*	This sub-theme describes the need for an actor (trustee) to be perceived as trustworthy and thus display characteristics of trustworthiness that are valued by another actor (trustor) in order to establish trust. It is discussed as a precursor to trust.	“As trustworthiness is strongly related to trust, it is a characteristic that researchers can develop to build trust within their partnership. Doing so requires understanding what the trustor (here, the community partner) cares about and considers valuable, and acting in a way that meets their expectations for the researcher’s motivation, process and outcome.” [47] “In addition to having a direct impact on trust, the perceived trustworthiness of the trustee also acts as a mediator between trust and several other factors included in our model.” [39]
*ST C2.2*	*Vulnerability*	This sub-theme speaks to the willingness of an actor (trustor) to be vulnerable to the actions of another actor (trustee). The trustor does not have complete control over how the trustee will behave and is thus, uncertain about how the individual will act, which also implies that there is something of importance to be lost, and in turn, risk involved. Therefore, in order to be vulnerable, there must be an opportunity for risk where the trustor must then decide if they are willing to take the risk of placing trust in the trustee. Furthermore, if there is the possibility of risk, this implies that there will be some level of uncertainty regarding how the trustee will behave. It is noted that if there is trust between partners, there is a lower level of uncertainty between how the trustee will behave. In summary, for this sub-theme we consider uncertainty and risk as necessary aspects of vulnerability.	“Moreover, trust entails being ‘vulnerable to the actions of another party based on the expectation that the other will perform a particular action important to the trustor, irrespective of the ability to monitor or control the other party’” [34] “Captured within these definitions of the trust relationship is the notion that having trust implies accepting a measure of exposure to risk.” [35] “that trust and mutual benefit allow both parties to share the risks that come from the uncertainty of unexpected occurrences that affect them both” [40]
*ST C2.3*	*Integrity*	This sub-theme concerns the extent to which the trustor thinks that the trustee will act in their best interest and the belief that the trustee will follow a set of principles, deemed acceptable by the trustor, such as they will say what is true.	“Seen as a relational phenomenon, trust describes taking another person’s ‘perspective into account when decision-making and not act[ing] in ways that violate the moral standard of the relationship’ [56]” [34] “trust was established through consistently fulfilling promises, attitudes of humility and caring” [48]
*ST C2.4*	*Reliability*	This sub-theme describes the confidence in and extent to which the trustor believes the trustee's will follow-through on commitments, perform a given task, and/or make decisions about something.	“Trust (the extent to which an organization was judged by other HIPMC members as being reliable in following through on commitments...)” [37]
*ST C2.5*	*Ability*	This sub-theme describes an individual’s (trustee) ability to perform a given task or make decisions about something based on their perceived skill set and competence from the perspective of another individual (trustor).	“Ability of the trustee, which refers to the skills and competencies of the trustee in a specific domain” [39]
*ST C2.6*	*Strength and quality of relationship*	This sub-theme explored the strength of a relationship with another individual and the quality of this relationship. For example, an acquaintance or a friend can describe a difference in the level and quality of the relationship.	“Trust often accompanies friendship and kinship, two of the core relations in every society. Trust sometimes accompanies working relations such as mentorship, advisory relations, or partnership. In many societies, trust accompanies multi-step relationships, such as friend-of-relative or mentor-of-friend. Whether affect-based trust, cognition-based trust” [54],
*ST C2.7*	*Shared values, vision, and goals*	This sub-theme highlights the need to have shared visions, values and goals in partnerships. Specifically, common goals, missions, and plans can promote trust.	“Trust comes in many forms, including based on a feeling of connectedness or shared values (affinitive)” [34] “Trust increases through the sharing of common goals” [48]
*ST C2.8*	*Power sharing + co-ownership*	This sub-theme explores sharing power, and fostering co-ownership in partnerships as a dimension of trust.	“Trust has four specific dimensions: (a) Supportive Environment; (b) Developing a Common Understanding; (c) Shared Power; and (d) Strategic Alignment of Group with Organization. Collective learning has five specific dimensions: (a) New Knowledge; (b)New Attitudes; (c)New Practices; (d) Problem Solving; and (e) Personal Concerns [43].
Theme C3	Complex concept	This theme emphasises some general features of trust identified across the literaure. Trust is discussed as a complex concept that is multidimensional, varying in conceptualisation across disciplines, and includes multiple types of trust.	
*ST C3.1*	*Multiplicities of trust*	The concept of trust has been defined as a variety of types depending on the strength and level of trust that exists between individuals, or whether the trust has been earned. Other trust types concern generalised trust; trust about people in general, or particularised trust; trusting a specific individual or group.	“generalized trust describes basic trust toward unspecified others in a society.” [50] “The trust typology was created as an alternative measure for understanding the process of trust development in CBPR partnerships [55]. This typology represents a developmental model, though not necessarily anchored at opposite poles.” [44]
*ST C3.2*	*Multidimensions of trust*	The lack of consensus surrounding a definition of trust speaks to its complexity as a concept. Specifically, it is not only a psychological phenomenon, and it can vary for each individual, across different social interactions, and across disciplines.	“Trust is an incredibly complex concept with many definitions and uses across several disciplines” [31] “Trust can be understood as a multidimensional” [52]
Theme C4	*Features of social network analysis*	This theme explores definitions of trust where it is defined in terms of its social network analysis properties.	
ST C4.1	*Reciprocal trust*	This sub-theme describes the presence of trust based on the notion that they think the trustee also trusts them back. Thus, if a trustor thinks that the trustee also trusts them, trust is thought (by the trustor) to be reciprocated (by the trustee).	In this study, we address trust at an individual (personal) level that refers, “to the extent to which individuals trust each other within the workplace (reciprocal trust).” [41]
ST C4.2	*Asymmetry*	This sub-theme describes trust as a concept where there is a “one-way” directional relationship between two individuals in a network. So individual “A” may have a relationship with individual “B”, but not B with A (or in the same capacity).	“In a dyadic trust relationship, most of the time, the trust relationship contains an asymmetry. Because of this asymmetry between the partners, one actor may take risks in trust relationships. This risk is a prerequisite of trust and it only exists in the context of decision and action.” [41]

*Legend: ST* sub-theme, *C(#)* conceptualisation of trust

#### Context-specific

This parent theme explores definitions of trust as a variable concept that is affected by the individuals in a given partnership and network. Indeed, individuals are unique in their disposition to trust, which is influenced by their personality and their experiences of trust, but also by the context *surrounding those individuals* such as the structural aspects including institutional barriers, norms, and values that surround trust:

First, initial trust depends on personality; people simply differ in their general disposition to trust/distrust [32].

trust must be understood from the perspective of all parties and within its context [44]

This notion that trust depends on context was widely discussed across the extracted literature (*n* = 18) [30, 32–35, 40, 41, 43–47, 49, 51–55], from both CBPR and social network focused studies.

#### Relational

All studies defined trust as a “relational” concept, involving a dyadic relationship where trust is being given by a trustor and received by a trustee. All but one of the extracted references [33] defined trust by mentioning at least one of the eight “relational” subthemes (see Table 3). This one study by Burt et al. [33] that did not mention one of the eight sub-themes did however discuss trust as a function of relationships, but strictly through a social network analysis lens, without further defining trust in regards to its relational features. Of the eight sub-themes discussed, integrity, reliability, and ability appeared to be closely related and thus were at times conceptually ambiguous across the literature. Therefore, we draw specific attention to their nuances as distinct concepts.

For instance, C2.3 “integrity” speaks to the actions of an individual from a moral or ethical perspective, such as whether or not the individual will act in the best interest of another individual:

Seen as a relational phenomenon, trust describes taking another person’s ‘perspective into account when decision-making and not act[ing] in ways that violate the moral standard of the relationship’ (Weber and Carter 1998, 3) [34].

Meanwhile C2.4 “reliability”, embodies some such aspects, but speaks more to the confidence the trustor has that the trustee will follow through on a commitment or perform a given task:

Trust (the extent to which an organization was judged by other HIPMC members as being reliable in following through on commitments...) [37]

Lastly, although C2.5 “ability” can also influence whether or not an individual performs a given task or is reliable, it speaks more to the perceived skillset and thus competence that the trustor feels the trustee has:

Ability of the trustee, which refers to the skills and competencies of the trustee in a specific domain [39]

Finally, there were two differences identified when exploring the presence of certain sub-themes for conceptualisation across CBPR and social network studies. Indeed, the sub-theme C2.6 “strength and quality of relationship” was only identified in social network studies [30, 41, 52, 54], while C2.8 “power sharing and co-ownership” was only recognized in CBPR studies [43, 45, 47].

#### Complex concept

As demonstrated by the three relational sub-themes discussed above (integrity, reliability, and ability), trust embodies distinct concepts within itself that could be conceptualised differently across the literature and thus, further explored and unpacked. Indeed, our third parent theme speaks to just this: trust as a complex concept. Specifically, trust was defined as a multiplicity of types that varied depending on strength and level, and/or who the trust was directed at, such as a general or particular population:

generalized trust describes basic trust toward unspecified others in a society [51].

The trust typology was created as an alternative measure for understanding the process of trust development in CBPR partnerships [55]. This typology represents a developmental model, though not necessarily anchored at opposite poles [45].

Trust can also be defined as multidimensional in that it differs across disciplines, social interactions and is not solely a psychological phenomenon. Of the extracted literature, many of the studies (*n* = 19) [30–33, 35, 36, 38, 39, 41, 44, 45, 47, 49–55] defined trust as a complex, multidimensional concept:

Trust is an incredibly complex concept with many definitions and uses across several disciplines [31]

Trust can be understood as a multidimensional [52]

Both sub-themes C3.1 “multiplicity of trust” and C3.2 “multidimensions of trust” were identified in the references extracted with a social network focus [30–33, 35, 36, 38, 39, 41, 49–54], as well as those with a CBPR focus [36, 44, 45, 47, 55].

#### Features of social network analysis

A final theme that was less common, but emerged across three social network studies, was conceptualising trust in regards to specific features of social network analysis [35, 41, 53]. Specifically, the social network analysis features used to define trust speak to the direction of receiving and giving trust, as either bi-directional (reciprocal) or unidirectional (asymmetric). Gursakal et al. [41] defined trust as reciprocal, but then discussed how trust relationships often contain some asymmetry, depending on context:

In this study, we address trust at an individual (personal) level that refers, “to the extent to which individuals trust each other within the workplace (reciprocal trust) [41].

In a dyadic trust relationship, most of the time, the trust relationship contains an asymmetry. Because of this asymmetry between the partners, one actor may take risks in trust relationships. This risk is a prerequisite of trust and it only exists in the context of decision and action [41].

Not surprisingly, only social network focused studies conceptualised trust in terms of their features of social network analysis. Specifically, three studies [35, 41, 53] identified the sub-theme C4.1 “reciprocal trust”, while one study [41] discussed C4.2 “asymmetry” in their conceptualisation of trust.

### Trust: Operationalised

The questions and indicators used to operationalise trust were coded into the same four emergent parent themes identified for the conceptualisation of trust (see Fig. 3). The sub-themes for operationalisation describe the questions and indicators of trust (for a complete list of themes, sub-themes, and descriptions, please refer to Table 4).

**Table 4:** Findings for operationalisation of trust

Operationalisation: What are the dimensions and indicators used for trust? What questions were asked to represent trust?			
Themes and sub-themes		Description	References
**Theme O1**	**Context specific**	This theme speaks to questions and indicators that ask about trust in a specific context. Questions and indicators pertaining to the context vary depending on the traits that exist within an individual as a kind of precondition to trust, as well as the context surrounding an individual, such as individuals in their network.	
***ST O1.1***	*Within individuals*	This sub-theme describes questions and indicators that explore how individuals within a context, and thus the traits that exist within the individual, can impact trust. Specifically, questions or indicators for trust that are dependent on the individual’s personality and experiences can alter their disposition to trust. For example, trust can be influenced by their past experiences with trust (or mistrust) in others (i.e. groups, individuals, and organisations).	“a. Talk to me about how you view trust within the POPS-CAB. I’m interested in hearing your views on benefits/opportunities as well as the challenges/barriers related to trust.” [46]
***ST O1.2***	*Surrounding individuals*	This sub-theme describes questions and indicators that explore how the context surrounding an individual influences trust. For instance, questions and indicators that discuss the norms, values, setting, institutional barriers, and level of support from others surrounding the individual in a given environment can represent trust.	“The trust network was measured by asking participants with respect to their particular team “Who do you trust?” [50]
**Theme O2**	**Relational**	This theme focuses on questions and indicators that speak to the relational aspects of trust highlighting its fluidity as a concept, while exploring a variety of features of trust that require and depend on another individual (i.e. trustor to trustee).	
***ST O2.1***	*Trustworthiness*	This sub-theme pertains to questions that explore an actor’s trustworthiness. This could include asking individuals, such as in a partnership, what they deem are important characteristics of trustworthiness, or more directly asking if an actor thinks another actor is trustworthy.	“The open-ended interview guide addressed each participants’ concept of trustworthiness, which actions or factors demonstrate a researcher’s trustworthiness within a particular partnership, what kinds of institutional barriers or facilitators influenced their experiences of trustworthiness within the partnership, and how the partnership has overcome any challenges to trust. Community partners were asked about characteristics of their academic partners; academic partners were asked about their trustworthiness and that of other researchers, and bridge partners were asked about academic partners and their sense of trustworthiness and responsibility in their unique roles.” [47]
***ST O2.2***	*Vulnerability*	This sub-theme explores questions that reflect an individual’s vulnerability to another, such as disclosing something about oneself.	“Would you tell [Student X] something personal about yourself? (Disclosure)” [34]
***ST O2.3***	*Integrity*	This sub-theme represents questions that ask about the extent to which the trustor thinks that the trustee will act in their best interest and the belief that the trustee will follow a set of principles, deemed acceptable by the trustor, such as they will say what is true.	“How often does [Student X] keep promises? (Promises)” [34] “Please indicate those who you think will act in your best interests” [39]
***ST O2.4***	*Reliability*	This sub-theme pertains to questions that ask about someone or a group of people's reliability. Reliability is discussed as an actor's ability to meet certain demands, perform specific tasks and make decisions. Reliability is reflective of one's competence from the perspective of the trustor-trustee dynamic. Specifically, it is related to the confidence in and extent to which the trustor believes the trustee will follow through on commitments, perform a given task, and/or make decisions about something.	“Most of my workmates can be relied upon to do as they say they will do.” [53]
***ST O2.5***	*Ability*	This sub-theme explores questions and indicators that describe an individual’s (trustee) ability to perform a given task or make decisions about something based on their perceived skillset and competence from the perspective of another individual (trustor).	“My co-workers are very capable of performing their job.” [41] “I feel confident about my co-workers’ skills” [41]
***ST O2.6***	*Strength and quality of relationship*	This sub-theme represents questions that ask about the strength and quality of a relationship with another individual.	“(2) On a scale of 1–10 (with 1 being ‘not good at all’ to 10 being ‘best friend’), how good of a friend is [Student X]?” [34]
***ST O2.7***	*Shared vision, values, and goals*	This sub-theme describes indicators and questions about shared visions, values and power in partnerships when operationalising trust.	“Total Trust: Average of the ranking given by all other members for that organization along three dimensions: reliability, support of mission, and open to discussion.” [37]
***ST O2.8***	*Power sharing + co-ownership*	This sub-theme explores sharing power, and fostering co-ownership in partnerships as an indicator used to operationalise trust.	“66. Shared power and decision making—acknowledge, minimize or address perceived power differentials and imbalances” [44]
**Theme O3**	**Complex concept**	This theme emphasizes indicators used to operationalise trust that speak to trust as a complex concept. Specifically, this includes indicators that explore trust as multiple types.	
***ST O3.1***	*Multiplicities of trust*	This sub-theme speaks to the indicators and questions that address specific types of trust depending on the strength and level of trust that exists between individuals, or whether the trust has been earned. These types could vary in strength such as no trust, neutral trust, to critical reflexive trust.	“Survey participants were asked to select the trust type they experienced at the beginning of their partnership and the type they currently experience.” [55]
**Theme O4**	**Features of social network analysis**	This theme explores indicators or questions that operationalise trust through social network analysis techniques and concepts.	
***ST O4.1***	*Reciprocal trust*	These questions ask individuals if they think that the trustee also trusts them, is it mutual?	“Do you think [Student X] trusts you? (only School 2)” [34] “SNA questionnaire for personal trust at intra organisational level measures personal trust levels of the co-workers to each other (reciprocal trust).” [41]
***ST O4.2***	*Homophily*	This sub-theme represents indicators that describe how people associate with individuals that share similar beliefs and values.	“Value homophily is based on the following two individual attributes: trust in peers and trust in management.” [53]
***ST O4.3***	*Structural equivalence*	Grouping of nodes in a network based on patterns of their connections to others in the network.	“We argue that structural equivalence represents a very useful construct for capturing how dyad members’ relationships with their entire constellation of third parties predicts their trust in one another.” [42]
***ST O4.4***	*Network closure*	Number of third parties to a relationship.	“We identify three distinct ways in which an employee and co-worker may be linked to third parties, each of which captures a different theoretical mechanism for influencing interpersonal trust. We refer to these as network closure, structural equivalence, and trust transferability (the names are derived from concepts in the network literature).” [42]
***ST O4.5***	*Transferability*	The number of third parties who trust the trustee and are also trusted by the trustor.	“And we explore how trust transferability may predict trust directly by conveying trust-related judgments from third parties to employees (see Fig. 1).” [42]

*Legend:ST* sub-theme, *O(#)* operationalisation of trust

#### Context specific

There are two sub-themes (O1.1 “within individuals” and O1.2 “surrounding individuals”) attached to the parent theme O1 “context-specific”. “Within individuals” describes the questions and indicators that explore how individuals within a context can impact trust, such as an individual’s unique disposition to trust:

a. Talk to me about how you view trust within the POPS-CAB. I’m interested in hearing your views on benefits/opportunities as well as the challenges/barriers related to trust [46].

Meanwhile, “surrounding individuals”, looks at questions and indicators that explore trust based on those surrounding the individual in a specific environment or network:

The trust network was measured by asking participants with respect to their particular team “Who do you trust? [50]

Both sub-themes were identified in social network [30, 34–36, 39, 42, 49–53] and CBPR [36, 43–48, 55] focused literature.

#### Relational

All of the eight sub-themes for the parent theme O2 “relational” mirrored that of how trust was conceptualised. For example, it is possible to identify the nuances between some relational sub-themes that were discussed earlier as conceptually ambiguous, such as: O2.3 “integrity”, O2.4 “reliability”, and O2.5 “ability”, by looking at the specific questions and indicators used to operationalise trust for each of these sub-themes. For example, O2.3 “integrity” was represented by questions and indicators that explore the extent to which a trustor thinks the trustor will act in their best interest:

Please indicate those who you think will act in your best interests [39]

While O2.4 “reliability”, speaks to the confidence that the trustor has in the trustee following through on commitments:

Most of my workmates can be relied upon to do as they say they will do [53]

And O2.5 “ability” captures questions and indicators that explore the trustee’s skillset from the perspective of the trustor:

I feel confident about my co-workers’ skills [41]

Two differences were observed when looking at the presence of sub-themes across social network and CBPR focused literature. Specifically, only social network-focused studies appeared to operationalise trust as O2.2 “vulnerability” [32, 34, 41]. However, a CBPR focused study by West K [47], did operationalise vulnerability in regards to trustworthiness, but not as trust specifically. Furthermore, similar to conceptualisation, two CBPR focused references [43, 44] operationalised trust as O2.8 “power sharing and co-ownership”.

#### Complex concept

For this parent-theme O3 “complex concept”, we noticed that one of the sub-themes discussed in conceptualisation, “multidimensions of trust”, was not captured in how trust was operationalised throughout the extracted literature. Specifically, there was only the one sub-theme, O3.1 “multiplicities of trust” identified. The “multiplicities of trust” represent questions and indicators that address specific types of trust:

Survey participants were asked to select the trust type they experienced at the beginning of their partnership and the type they currently experience [55].

Multiplicities of trust was identified in both social network [41, 51] and CBPR [45, 55] studies.

### Features of social network analysis

This parent theme is where we see most of the variation in sub-themes compared to how trust was conceptualised. Moreover, the only consistent sub-theme across how trust was conceptualised and operationalised is O4.1 “reciprocal trust”:

SNA questionnaire for personal trust at intra organisational level measures personal trust levels of the co-workers to each other (reciprocal trust) [41].

Meanwhile, four new sub-themes were presented for how trust was operationalised: O4.2 “homophily”, O4.3 “structural equivalence”, O4.4 “network closure”, and O4.5 “transferability”.

This presence of more features of social network analysis used to operationalise trust compared to conceptualise trust is less surprising given the less abstract and more practical nature of operationalisation.

### Trust: Measurement

How trust was measured across the extracted literature was organized into two main parent themes, M1 “type of measurement” and M2 “level of measurement”. Each of the parent themes had four sub-themes: M1.1 “survey”, M1.2 “scaling”, M1.3 “qualitative”, and M1.4 “unobtrusive” and M2.1 “nominal”, M2.2 “ordinal”, M2.3 “open-ended questions”, and M2.4 “ratio” (for a complete list of themes, sub-themes, and descriptions, please refer to Table 5).

**Table 5:** Findings for measurement of trust

Measurement: how is trust measured?			
Themes and sub-themes		Description	References
**Theme M1**	**Type of measure**	What type of measures was (survey, scaling, qualitative, unobtrusive) used for trust?	
***ST M1.1***	***Survey***	The type of measurement used to measure trust was a survey.	“we drew upon previously validated survey instruments used to measure peer-to-peer trust in classroom settings” [34] “The web-based survey provided the types of trust with their definitions” [45]
***ST M1.2***	***Scaling***	A scale was used to measure trust.	“The respondents were also asked to rate the level of trust they have that they will be provided with the input they need from each identified other actor (on a similar Likert scale from no trust to full trust)” [31]
***ST M1.3***	***Qualitative***	The type of measurement technique used to measure trust was qualitative.	“The question of trust often led to open-ended responses which were recorded and probed on.” [36] “In-depth interviews were conducted between October 2015 and September 2016, by phone (*n* = 28) and in-person (*n* = 3).” [47]
***ST M1.4***	***Unobtrusive***	The type of measurement technique used was unobtrusive and thus does not require the researcher to intrude in the research context.	“Observations in the US and especially in Malawi helped me understand the context and day to day challenges in Malawi (see Table 4.4 for a description of observations in Malawi).” [48]
**Theme M2**	**Level of measure**	What level of measurement was used (nominal, ordinal, interval, ratio) to measure trust?	
***ST M2.1***	***Nominal***	Items are named, but are in no specific order. The numbers assigned to it are thus arbitrary.	“TRUST: 0 = Did not select respondent 1 = Selected respondent” “Asked participants to select the most appropriate type of trust at the beginning of their partnership and the current stage of their partnership and to choose the type of trust expected in the future.” [45]
***ST M2.2***	***Ordinal***	Items can be ordered, such as level of agreement, of low to high degrees of trust.	“Scale from 1–4 one being ‘poor relationship/little trust’ and four being “excellent relationship/high trust” [35] “The scale consisted of self-report items scaled in a five-point Likert scale (1 = completely disagree to 5 = completely agree).” [41]
***ST M2.3***	***Open-ended question***	There was no forced choice for these questions.	“65. What could be done to improve the trust among movement members?” [49]
***ST M2.4***	***Ratio***	Items are named, but are in no specific order. The numbers assigned to it are thus arbitrary.	“Percentage, A 100 % occurs when all members trust others at the highest level” [37]

*Legend ST* sub-theme, *M(#)* measurement of trust

#### Type of measurement

The type of measurement used across the literature often involved more than one type. This is not surprising given the mixed-method nature of many of the studies [30, 31, 34, 36, 40, 44–49, 51, 52, 55]. All but two of the studies involved the administration of a survey [44, 48] and all but one of the studies incorporated scaling questions [43, 48]. MacIntyre et al. [48] did not incorporate scaling questions, but conducted structured interviews and observation. Finally, only one study by Ardoin et al. [33] incorporated all four types of measurement in their study design.

#### Level of measurement

The level of measurement was somewhat consistent across studies. For instance, all but five studies [39, 43, 45, 48, 50, 52] incorporated an ordinal level of measurement in their study to measure trust. Six studies [39, 43, 45, 50, 52, 55] included a nominal level of measurement, and only one study by McCullough et al. [37] incorporated a ratio level of measurement, but also included an ordinal level of measurement in the study. Finally, four studies [43, 44, 48, 49] incorporated open-ended questions.

When comparing type and level of measurement across social network and CBPR focused literature, no apparent patterns were observed.

### Trust: Outcomes

Studies were also coded by their outcomes, exploring study findings that were identified to be associated with trust in some manner. By coding the outcomes, we could more easily identify patterns across and within studies. Mirroring that of conceptualisation and operationalisation, there were four parent themes identified for the outcomes: R1 “context specific”, R2 “relational”, R3 “complex concept”, and R4 “features of social network analysis”.

Comparatively with conceptualisation and operationalisation, the same sub-themes were identified for R1 “context-specific”, and similar to operationalisation, there was one sub-theme identified for R3 “complex concept”. Interestingly, however, R2 “relational” and R4 “features of social network analysis” saw new sub-themes emerge in the outcomes. For example, for R2 “relational”, there were three additional sub-themes identified, while for R4 “features of social network analysis”, 11 new sub-themes emerged as outcomes associated with trust (see Fig. 3 for a complete list of sub-themes and Table 6 for a list of parent and sub-theme descriptions).

**Table 6:** Findings for outcomes pertaining to trust

Outcomes: What were the outcomes of the study?			
Themes and sub-themes		Description	
**Theme R1**	**Context specific**	This theme describes outcomes of trust that are influenced by given context. Specifically, outcomes that describe how/if context varies depending on the traits that exist within an individual as a kind of precondition to trust, as well as the context surrounding an individual, such as individuals in their network.	
***ST R1.1***	***Within individuals***	This sub-theme describes outcomes that explore how an individual within a context, and thus the traits that exist within the individual can impact trust. Specifically, trust is discussed as dependent on the individuals personality and experience which can alter their disposition to trust. For example, trust can be influenced by their past experiences with trust (or mistrust) in others (i.e. groups, individuals, organisations etc.).	“Although we were not able to assess the impact of a trustor’s propensity to trust as part of our net logit model, a careful analysis of the difference between the actual and predicted values suggests that propensity to trust is another key variable influencing interpersonal trust in networks.” [39]
***ST R1.2***	***Surrounding individuals***	This sub-theme describes outcomes that explore how the context surrounding an individual can influence trust. For instance, the norms, values, setting, institutional barriers, and level of support from others surrounding the individual in a given environment can influence trust.	“A third finding relates to the notion that some universal dimensions of trust are important in the classroom, as well as in the field-based setting, while other domains may be more distinctive for residential, field-based experiences...This finding suggests that these may be distinct notions of trust particularly pertinent to this field-based setting.” [34]
**Theme R2**	**Relational**	This theme focuses on outcomes that speak to the relational aspects of trust and highlight the how trust is fluid from a relational perspective and involves a variety of features that require and depend on another individual (ie., trustor to trustee).	
***ST R2.1***	***Trustworthiness***	This sub-theme explores outcomes of trustworthiness, which has been described as a precursor to trust.	“For all three measures of perceived trustworthiness—Expertise, Interest, and Values—a positive and significant relationship exists with Trust.” [39] “Based on data from community, academic, and bridge partners, I identify four major dimensions of trustworthiness in the community-academic research partnership setting: ethical, competent, caring, and vulnerable. Each dimension has several subthemes, and a cross-cutting theme, respectful” [47]
***ST R2.2***	***Cohesion***	This sub-theme pertains to outcomes that explore how communication, collaboration, cooperation, and coordination function to create a cohesive partnerships and/or teams where people can work together effectively, thus promoting trust.	“Our results suggest that when respondents indicated a high level of trust in their linkages with other organisations, regardless of which sector organisations belonged, they were more likely to collaborate.” [35] “As a means to create a strategic competitive advantage for CSOs, trust holds promise as a means to enhance administrative coordination in local networks, access resources, and create the means to cooperate with those in the environment in which they are embedded.” [35] “Collaboration and cooperation among CAB members: Members from different organizations collaborate to solve problems and cooperate to share resources and responsibilities in a manner that encourages trust that tasks will be completed (Trust; Problem assessment; Resources; Group roles)” [46]
***ST R2.3***	***Relationship quality and relationship type***	This sub-theme pertains to outcomes that explore how the quality of a relationship, and type, whether it be a friend or another type of personal relationship, are correlated with trust.	“TRUST: Trust correlates very strongly with the type of relationship the actors have with each other (0.9314), meaning that the better friends they are, the more trust they express.” [31] “This is not surprising considering the very strong correlation between trust and relationship.” [31] “It became clear through our comparison that quality of relationship, one measure of trust, varied across the networks.” [40]
***ST R2.4***	***Support***	This sub-theme reflects outcomes that discuss how support in general, such as moral or social support are correlated with trust.	“It is again the emotional input of pepping and moral support that has the highest relative importance for trust by far, but with a smaller gap to report of activities again on second place (Fig. 3).” [31] “This study demonstrated statistically significant relationships between social support and trust, as well as social support, participatory discussion, and participatory decision-making and coordination. However, unlike previous interorganizational network studies, statistically significant relationships were not found between conflict resolution, participatory discussion, or participatory decision-making and trust. ” [49]
***ST R2.5***	***Reliability***	This sub-theme pertains to outcomes that discuss how an individual or a group of individual’s are relied on to meet certain demands, perform specific tasks and make decisions is associated with trust. Reliability is reflective of one’s competence from the perspective of the trustor-trustee dynamic. Specifically, it related to the confidence in and extent to which the trustor believes the trustee's will follow-through on commitments, perform a given task, and/or make decisions about something.	“my study participants were clear that reliability was necessary for trustworthiness. It holds that if a person cannot be relied upon to keep their word, they cannot be trusted.” [47] “Question 65, What could be done to improve the trust among movement members?” “Each person does their own thing in their own way, make sure that you follow-through on your tasks (*N* = 2)” [49] “Listening to the community’s priorities, engaging the community in activities and sharing information with the community an NGO help build trust. Doing what is promised substantiates words with actions” [48]
***ST R2.6***	***Ability***	This sub-theme describes outcomes pertaining to an individual’s (trustee’s) ability to perform a given task or make decisions about something based on their perceived skill set and competence from the perspective of another individual (trustor).	“Recognition and sharing of expertise: Expertise is valued as a resource that provides legitimacy to the CAB, influences trust in member abilities, provides confidence in project success, defines group roles and responsibilities, and guides engagement and influence” [46] “H4: These separate main effects for receiving ties indicate that players high in performance are more likely to be trusted, and also players high in experience are more likely to be trusted by others. (significant)” [50]
***ST R2.7***	***Integrity***	This sub-theme represents outcomes that pertain to one’s belief that the trustee will follow a set of princples, deemed acceptable by the trustor. For example, questions may ask if an individual is likely to say what is true and share that with the trustor.	“Taken together, the results of the foregoing analysis corroborate the idea that trust is associated with deeper hierarchies of coherent level 2 and 3 beliefs. The analysis reveals not only a strong covariation among respondents’ level 2 and 3 beliefs about alters and their trust in the competence and integrity of alters, but also with their knowledge of alters’ level 1 beliefs.” [38] “There is no formulaic method for gaining community trust. Although participants have outlined major facilitators and barriers to trust, ultimately, relationships of trust with community members evolve when NGOs are respectful, do what they say they will do, and involve community members fully in all processes.” [48] “.862 My research partner really looks out for what is important to me.#” [47]
***ST R2.8***	***Shared values, visions, and goals***	This sub-theme reflects outcomes that highlight how having shared visions and goals in partnerships as well as a commitment to these partnerships, can promote trust in relationships.	“Commitment to a shared vision: There is a shared understanding of the childhood obesity problem in the region and commitment to finding solutions that encourages trust in following through on tasks and confidence in sustainability of efforts (Problem assessment; Trust; Sustainability)” [46] “Trust increases through the sharing of common goals and an ongoing commitment between individuals and organizations. Participants emphasized that the community must be ‘behind’ any program or project if it is to succeed.” [48] “This setting contains particular power dynamics, historical experiences, and differences in values and goals that set this relationship apart from other types of trust relationships. It demands a particular focus on respect as well as an additional dimension, vulnerability, that is often associated with trusting rather than trustworthiness.” [47]
***ST R2.9***	***Problem solving***	This sub-theme discusses outcomes that identify how problem solving in partnerships can lead to the development of trust in relationships.	“Question 65, What could be done to improve the trust among movement members?” “13. Reflect on new solutions to problems (*N* = 1)” [49] “The final five clusters representing factors that contribute to trust in community-academic research partnerships, were named as follows: 1) authentic, effective and transparent communication, 2) mutually respectful and reciprocal relationships, 3) sustainability, 4) committed partnerships and, 5) communication, credibility and methodology to anticipate and resolve problems.” [44]
***ST R2.10***	***Power sharing + co-ownership***	This sub-theme discusses outcomes that explore how having respect in a partnership, sharing power, and fostering co-ownership can promote trust. For example, including partners in decision-making, taking their perspectives into account throughout all stages of the research process, and sharing ownership of project tasks are essential for showing respect to partners, thus promoting trust in the relationship.	“This setting contains particular power dynamics, historical experiences, and differences in values and goals that set this relationship apart from other types of trust relationships. It demands a particular focus on respect as well as an additional dimension, vulnerability, that is often associated with trusting rather than trustworthiness.” [47] “The trusting environment was also associated in interviews with country ownership, which was in contrast to the rushed process and lack of ownership in the subsequent IPV application process.” [36] “Three main barriers to trust that can be addressed internally by an NGO were identified by participants: 1) NGO arrogance and assumptions; 2) Not obtaining community support for NGO activities; and 3) NGO activities and or research that benefits outsiders rather than the community. NGO arrogance and assumptions refer to a power differential whereby the NGO perceives itself as the expert and dismisses, denigrates or ignores community knowledge and expertise.” [48]
***ST R2.11***	***Sustainability***	This sub-theme discusses outcomes that pertain to the sustainability of partnerships or specific outcomes (such as a program) beyond project or funding end date.	“This relationship may also proceed in the inverse direction, such that increased sustainability will lead to increased trust and more collective learning” [43] “Barriers and facilitators to community trust were identified by participants and included respect for cultural norms, listening to community members and asking them about their priorities and involving community members in any project or research activity if sustainability is a goal.” [48]
***ST R2.12***	***Vulnerability***	This sub-theme speaks to outcomes of trust that focus on the willingness of an actor (trustor) to be vulnerable to the actions of another actor (trustee). The trustor does not have complete control over how the trustee will behave and is thus, uncertain about how the individual will act, which also implies that there is something of importance to be lost, and in turn, risk and uncertainty involved.	This setting contains particular power dynamics, historical experiences, and differences in values and goals that set this relationship apart from other types of trust relationships. It demands a particular focus on respect as well as an additional dimension, vulnerability, that is often associated with trusting rather than trustworthiness [47].
**Theme R3**	**Complex concept**	This theme explores outcomes of trust that speak to trust as a complex concept. Specifically, this includes outcomes that relate to the multidirectional nature of trust, including the multiple types of trust.	
***ST R3.1***	***Multiplicities of trust***	This sub-theme represents outcomes that explore trust not as a binary concept (presence/absence of trust), but in terms of types of trust. Depending on the type of trust present, the strength of trust will vary. For example, trust types can expand from (low) no trust, neutral trust, to (high) critical reflexive trust.	[“Both the qualitative and quantitative outcomes indicate that trust types do exist in practice. Data provided evidence that many partnerships began in mistrust/suspicion or proxy trust, and over time those same partnerships shifted to functional or critical reflective trust. In terms of unique contributions the qualitative data provided information about what contributed to the process of trust development” “Qualitative data elaborated on how and why types of trust developed over time, which showcased the transformative nature of CBPR. Third, through these data we see trust functioning at the local levels, with emerging patterns that can be transferable to other contexts.”] [45]
**Theme R4**	**Features of social network analysis**	This theme explores outcomes where social network analysis techniques were used to describe trust or how techniques are correlated with trust.	
***ST R4.1***	***Individual level***		
*ST R4.1.1*	*Constraint*	This sub-theme explores outcomes related to constraint. Constraint occurs when alters are connected to each other and can keep information from the ego and can therefore control ego’s actions and perceptions. Specifically, constraint measures the connections between alters from each of the alter's perspective.	“Among the network measures, each respondent’s constraint is positively correlated with her or his own level 2 belief strength, consistent with the idea that the density of relationships promotes interpersonal cohesion and formation of norms (e.g., Coleman 1988, 1990).” [38] “The final model shows that, again, structural hole theory seems to be the best predictor of the evolution of the trust network: individuals will not intensify their ties to persons who exert little structural constraint on them, and they will tend to initiate more trust relations the more efficient their network is. In sum, it seems that people strive after reciprocal trust relationships, and try to optimize their position within the network (i.e. search for the right mix of strong and weak ties).” [53]
*ST R4.1.2*	*Reciprocal trust*	Outcomes where the direction of tie goes both ways. For example, reciprocal trust is present if the trustor chooses to trust the trustee and the trustee also trusts the trustor.	[“The final five clusters representing factors that contribute to trust in community-academic research partnerships, were named as follows: 1) authentic, effective and transparent communication, 2) mutually respectful and reciprocal relationships, 3) sustainability, 4) committed partnerships and, 5) communication, credibility and methodology to anticipate and resolve problems.” [44] [“The reciprocity effect shows that there is a strong tendency to establish reciprocal trust relationships (*t* = 1.15/0.22 = 5.23; *p* < .001).” [53] “In all clubs, there is support for reciprocity of trust relations, and thus H1 relating to trust-generating mechanisms. It is no surprise that we found the significant presence of mutual trust ties in all three clubs, especially given that the reciprocal nature of trust is seen as fundamental to the definition of trust itself. Indeed, reciprocity can almost be seen as the fundamental structure for trust relations, and the absence of such patterns from a network of trust would indicate an incredible lack of trust within the network.” [50]
*ST R4.1.3*	*Asymmetry*	Outcomes where there is a “one-way” directional relationship between two individuals in a network. So the trustor may have a relationship with the trustee, but not the trustee with the trustor (or in the same capacity).	“Since more aging-in-place service transaction information is available and accessible by the public after desensitization, it could help eliminate the information asymmetry between older people and aging-in-place service centers, which could contribute to increasing the trust in service providers.” [30]
*ST R4.1.4*	*Centrality*	Outcomes pertaining to the extent to which a person inhabits a prestigious or critical position in a network.	“More strikingly, however, respondents’ strength of trust in and level 2 and 3 beliefs about alter j are negatively and significantly correlated with alter j’s betweenness centrality. These negative correlations are consistent with the idea that network centrality fosters a competitive orientation among actors as they attempt take advantage of opportunities for information brokerage and control to increase their autonomy and others’ dependence on them (Burt 1992, Moldoveanu et al. 2003).” [38] “H3: This is support for the third structure of trust (H3) and indicates that some highly popular teammates do not trust one another. (Significant for club A and B)” [50]
*ST R4.1.5*	*Transferability*	Outcomes exploring the number of third parties who trust the trustee and are also trusted by the trustor.	“Also contrary to our expectations, trust transferability is not a significant predictor of any of the three measures of a trustee’s perceived trustworthiness. However, Transferability is a positive and significant predictor of Trust directly.” [39] “We found that OCBIs and trust transferability had direct relationships with trust (Hypotheses 1 and 4)” [42]
*ST R4.1.6*	*Nodes-network members*	Outcomes pertaining to individual network members, representing the nodes in the network.	“Network members are highly trusted classmates and the ones with whom students actually do or would team up given the opportunity to self-select their team.” [32] “Two findings stand out: First, the connection between trust and social network is robust to most differences between individuals, especially business and political differences. Trust variance is 60% network context, and 10% individual differences” [33] “As the number of positive relationships between individuals from the community and NGOs increase, a ‘web of trust’ is developed.” [48] “It is important to note that the process of gaining community trust is built on individual dyads between an NGO staff member and a community member.” [48]
***ST R4.2***	***Group level***		
*ST R4.2.1*	*Cliques*	Outcomes that found a set of points all directly connected to each other	“The results (not shown) support Simmelian tie theory: the more cliques ego belongs to, the less likely it is that ego will initiate trust relationships to new alters, unless ego and alter are strongly tied to each other by both being member of the same cliques” [53]
*ST R4.2.2*	*Fragmentation*	Outcomes that found the proportion of pairs of nodes that cannot reach each other.	“With this study, fragmentation scores were relatively low meaning that many ties were realised, suggesting that the “coordinator” role has facilitated the development of many ties between organisations in the network.” [35]
*ST R4.2.3*	*Structural equivalence*	Outcomes where the grouping of nodes in a network are based on patterns of their connections to others in the network.	“Furthermore, our analysis suggests that although we did not find empirical evidence supporting Hypotheses 4, 8, and 9, frequency of interactions, structural equivalence, and trust transferability do influence the development of interpersonal trust. These variables just do not affect the development of interpersonal trust through the pathways that we originally hypothesized. Instead of influencing a trustee’s perceived trustworthiness, frequency of interactions between the trustor and trustee has a direct, positive impact on whether the trustor trusts the trustee.” [39] “But unlike network closure, Equivalence has a stronger direct effect on the three trustworthiness variables than on successful past cooperation. Based on these results, past cooperation does not mediate the relationship between structural equivalence and perceived trustworthiness. Instead, structural equivalence appears to have a direct, positive impact on whether a trustor perceives a trustee as trustworthy.” [39] “We found that structural equivalence predicted trust indirectly via OCBIs.” [42] [interpersonal organizational citizenship behaviors (OCBIs)]
*ST R4.2.4*	*Third party relationships*	This sub-theme discusses outcomes that show how trust was correlated with third-party relationships. Third-party relationships refer to when an actor in a dyad (two actors) goes outside the dyad to an additional third-party that is thus outside of the dyad to make decisions about trust pertaining to the other actor in the dyad.	“In other words, as the number of third parties who trust the trustee and are also trusted by the trustor increases, the likelihood that the trustor will trust the trustee increases.” [39] “Third-party relationships as a force that influences trust by shaping interpersonal behavior.” [42]
***ST R4.3***	***Network level***		
*ST R4.3.1*	*Network size*	This sub-theme pertains to outcomes that looked at the size of the network, such as how many actors/individuals make up the given network.	“The results show that guanxi ties are less distinct in larger, more open networks. With respect to network size, trust is higher in bridge relations with nonevent contacts (4.67 *t*-test in Table 4), and less increased for event contacts (− 4.27 *t*-test). There is no change in the closure-trust association.” [33]
*ST R4.3.2*	*Structural holes*	Outcomes pertaining to structural holes, which is where a lack of direct contact or ties between two or more entities (Burt, 1992).	“The final model shows that, again, structural hole theory seems to be the best predictor of the evolution of the trust network” [53]
*ST R4.3.3*	*Closure*	Outcomes pertaining to the number of third parties to a relationship (i.e. dense clusters of strong connections).	“Trust increases within a relationship as network closure increases around the relationship, but some relationships mature into guanxi ties within which trust is high and relatively independent of the surrounding social structure.” [33] [“H2: Second, concerning the functional equivalence perspective, network closure was positively related to network trust in both countries. Specifically, all three indicators of network closure were related to network trust in SC-China, while two of them (i.e. proportion of family ties and average closeness) were associated in SC-USA. These results support H2. H3 + H4: H3 that predicted a negative relationship between network closure and generalized trust is thus rejected [51]. Third, in regard to the mutual independence perspective, the single factor of individual network diversity and resources is negatively related to network trust in both nations as supposed by H4.” [51]
*ST R4.3.4*	*Homophily*	This sub-theme explores research outcomes where the study found that trust was stronger when people were interacting with those who were similar to them (e.g., had similar networks to them, or were from the same organisation or sector) when compared to those who were dissimilar to them (e.g., from different organisations of sectors, or had different networks).	“Interestingly, the results do not support the notion of homophily in this study as organisations were not likely to seek out collaborations with only those organisations in the same sector.” [35] “This suggests that individuals are more likely to perceive individuals who have similar social networks as trustworthy regardless of whether they have successfully cooperated in the past.” [39] Trust relations will be afforded to other team members of similar experience and performance. For Club A there is a significant and positive homophily effect for experience [.006 (.002)*], indicating that players trust others of a similar level of experience to themselves (supporting H5a) [50].
*ST R4.3.5*	*Density*	Outcomes that are based on the number of connections in a network.	“Density is the opposite: Trust is lower in bridge relations with nonevent contacts (− 8.23 *t*-test in Table 4), and increased for bridge relations with event contacts (9.33 *t*-test).” [33] “The high level of trust within the HIPMC coalition represents a critical strategic asset for network success. Trust can be challenging to build within a network. In its absence, efforts to optimize density and centralization may face meaningful barriers. Sustaining high levels of trust should become a key priority for coalition leaders moving forward.” [37] “Among the network measures, each respondent’s constraint is positively correlated with her or his own level 2 belief strength, consistent with the idea that the density of relationships promotes interpersonal cohesion and formation of norms (e.g., Coleman 1988, 1990).” [38]
*ST R4.3.6*	*Centralisation*	Outcomes looking at the degree to which network ties are focused on one individual, or a set of individuals.	“The results include a degree centralisation measure of 0.76 for collaborative ties and 0.77 for trust, suggesting that centrality and power are concentrated among a few organisations rather than dispersed across several organisations in both matrices.” [35] [The high level of trust within the HIPMC coalition represents a critical strategic asset for network success. Trust can be challenging to build within a network. In its absence, efforts to optimize density and centralization may face meaningful barriers. Sustaining high levels of trust should become a key priority for coalition leaders moving forward.” [37]

*Legend: ST* sub-theme, *R(#)* outcome pertaining to trust

Similar to conceptualisation and operationalisation, R2 “relational” features of trust continued to be the most common parent theme for outcomes related to trust across studies. Comparatively, the second most prevalent parent theme was, R4 “features of social network analysis”, indicating the reporting of more features of social network analysis connected with trust in some way (e.g., associated or indicating trust).

When looking at the differences in sub-themes across social network and CBPR literature, similar to operationalisation, we see the majority of features of social network analysis sub-themes emerging in social network focused literature. However, we did identify one study by Dave et al. [44] which was CBPR focused, that discussed outcomes pertaining to reciprocal trust. Furthermore, a study by McCullough et al. [37] that had both a social network and CBPR focus, discussed centralisation as an outcome from a trust network.

## Discussion

In summary, when exploring all three concepts together (trust, CBPR and social networks), we identified 26 references that met our inclusion criteria, with an overwhelming majority exploring trust in social networks. Following, an iterative and in-depth analysis of this literature occurred, which provided clarification for how trust was conceptualised, operationalised, and measured. Furthermore, our thematic analysis revealed an emergent category that highlighted another important dimension of trust—outcomes pertaining to trust. Interestingly, the same four parent themes; context-specific, relational, complex concept, and features of social network analysis, emerged for how trust was conceptualised, operationalised and outcomes pertaining to trust. This was not consistent for measurement of trust, due to the nature of the category, in that it involved the level and type of measurements used in the literature. Furthermore, no key patterns were shown for how trust was measured based on social network or CBPR focused literature. Indeed, sub-themes that emerged were also similar across conceptualisation, operationalisation and outcomes pertaining to trust. The primary differences in how the literature conceptualised and operationalised trust, and the outcomes pertaining to trust can be recognised at a sub-theme level. In general, it seemed that more features of social network analysis emerged when the literature operationalised trust, and even more so when they discussed outcomes of trust. Finally, when exploring the dimensions of trust across CBPR and social network literature, we saw an intersection in many of the themes and sub-themes that emerged, while noting only a few differences. For example, for conceptualisation, the sub-theme C2.6 “strength and quality of relationship” was only present in social network-focused literature [30, 41, 52, 54], while C2.8 “power sharing and co-ownership” was only present in CBPR focused literature [43, 45, 47]. As for operationalisation, the sub-theme O2.8 “vulnerability” was only discussed in social network-focused literature [32, 34, 41], while again, O2.8 “power sharing and co-ownership” was only mentioned in CBPR literature [43, 44].

As the first scoping review exploring all three concepts (trust, CBPR, and social networks) together, this research adds to the existing literature in a few key ways. First, the analysis from this scoping review illuminates the complexities of trust. Second, the analysis highlights the variation within studies in how they conceptualise and operationalise trust, as well as the outcomes of trust. Finally, this research provides important insight into the multidimensionality of which trust operates as a context, mechanism, and outcome.

Recognizing these connections with existing literature, the findings from our scoping review identify important implications for future research. First, by illuminating the complexities of trust, future research in the field of CBPR may be better positioned to strengthen the conceptual rigour and consistency of trust in their own CBPR studies. For example, within most individual studies, trust was operationalised differently than it was conceptualised, even when only looking at parent themes. Specifically, only four studies were coded with the same parent themes for how they conceptualised and operationalised trust [37, 41, 43, 55]. For example, a study by Neu, W [32] conceptualised trust comprehensively incorporating context, relational and complex concept into their conceptualisation of trust, while only tapping into the relational features when operationalising trust. Comparatively, Ferrin et al. [42] conceptualised trust only in terms of its relational features, but operationalised trust more comprehensively, including context-specific, relational, and features of social network analysis for the questions and indicators used.

Second, it would be valuable for future research to consider the issue of multidimensionality within partnerships and specifically appraise trust to see if it is operating as a context, mechanism and outcome, and is thus compatible with the realist perspective posited by Jagosh et al. [8] and further developed in Jagosh et al. [9] Indeed, our paper expands on this seminal work that understood trust to be a foundational element of partnership synergy but never further explored what the specific dimensions of trust were. Thus, our now enhanced understanding of trust as a context, mechanism, and outcome, affords researchers the opportunity to incorporate this knowledge to better understand why theories such as partnership synergy could lead to better partnership outcomes.

Finally, this review provides scope for prospective, longitudinal research to investigate and support trust in partnerships by paying specific attention to the multidimensionality of trust and thus identifying ways to improve trust as appropriate. For example, our enhanced understanding of trust reinforces the notion that trust, CBPR, and social networks constitute a conceptual triad, which is a valuable way to explore how partnerships can lead to better research outcomes. For example, in the CBPR conceptual model [2, 5] power dynamics are an important part of both the context and partnership process and are linked to trust in partnerships [57]. Using our enhanced understanding of trust, it may be possible to identify where power dynamics exist by identifying where asymmetrical trust relationships (a social network feature) are within the network. This shows the usefulness of exploring CBPR through a social-relational lens: network techniques can be employed to operationalise and measure the process and mechanisms that lead to success in CBPR.

### Limitations

Although findings from this scoping review present an important perspective for which to approach future research, some limitations should be considered. As illustrated in the findings, trust is a complex concept that contains specific features and attributes that themselves are complex and could be further explored. With this in mind, we brought our perspective and understanding when interpreting themes throughout the literature, which may vary from how others interpret findings from the extracted literature included in this review. However, we tried to ensure rigour in this review by continuously engaging in discussions amongst each other to consider interpretations of the data from multiple perspectives. We also made use of a reflexive research journal, as suggested by Braun and Clarke [28], which incorporated thoughts and decisions made throughout the review, while exploring how our assumptions may have impacted reported themes. Another limitation is a lack of public and patient involvement, which is an optional additional stage according to Arksey and O’Malley [23], in the interpretation of our review results that may have added some additional insight to our findings. However, as this scoping review is the first stage in a larger collaborative research process, further consultation will take place with these and other stakeholders at subsequent stages of the project. Finally, some dynamics of trust may have been missed as the refined inclusion criteria incorporated only participatory *health* partnerships, as opposed to other kinds of community partnerships.

## Conclusion

In conclusion, findings from this scoping review provide a comprehensive overview of how trust was conceptualised, operationalised, and measured and the outcomes of trust throughout social network and CBPR literature. Although there are important considerations to address when conducting research in this area, such as the complexity of trust as a concept, findings provide support for future research to incorporate trust as a lens to explore the social-relational aspects of partnerships.

## Supplementary Information

**Additional file 1.** CINAHL search strategy.

**Additional file 2.** PRISMA-ScR checklist.

**Additional file 3.** Individual study findings from the extracted literature.

## Data Availability

The dataset used and analysed during the current study is available from the corresponding author on reasonable request.

## Abbreviations

PR: Participatory research
CBPR: Community-based participatory research
SN: Social network
SNA: Social network analysis
