# Supplementary figures and images for: Conceptualising, operationalising, and measuring trust in participatory health research networks: a scoping review
Source: Syst Rev. 2022 Mar 6;11:40. doi: 10.1186/s13643-022-01910-x (PMC8900447; doi:10.1186/s13643-022-01910-x)

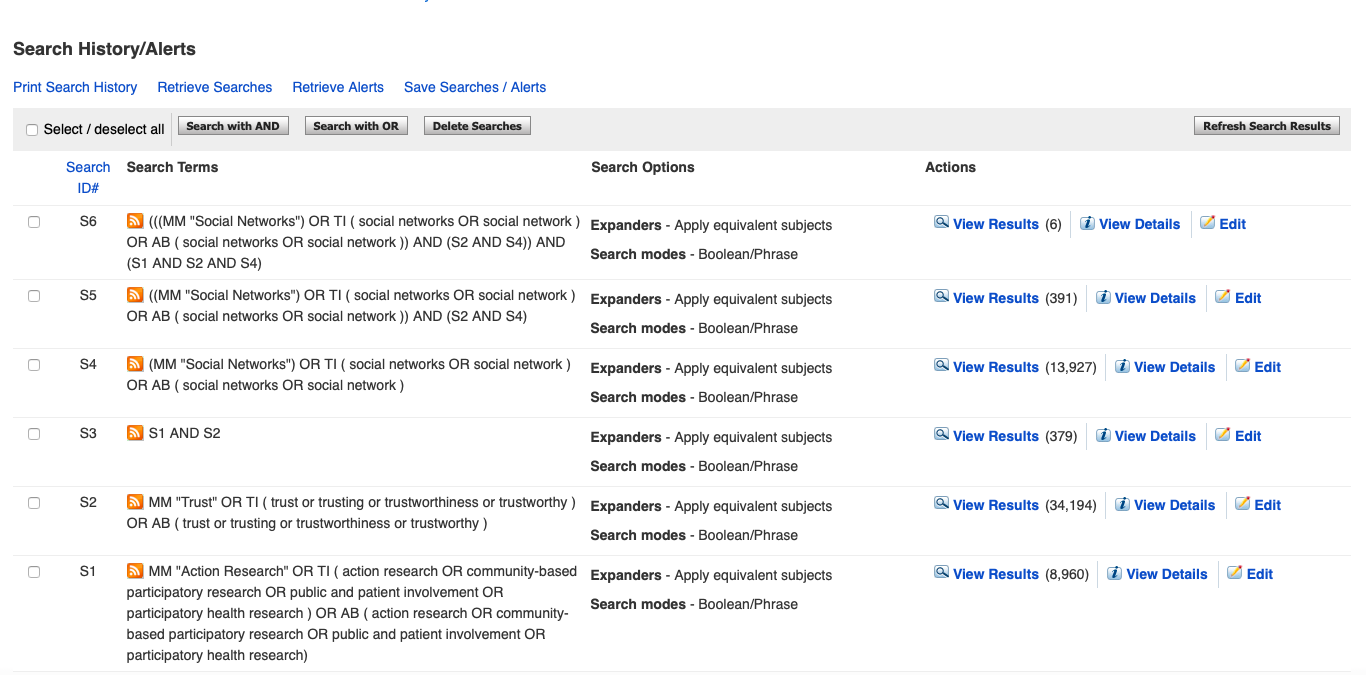
**Additional File 1** CINAHL Search Strategy

Supplement: Supplementary file 1 — Additional file 1. CINAHL search strategy. [file 13643_2022_1910_MOESM1_ESM.docx]
