# Supplementary material for: Conceptualising, operationalising, and measuring trust in participatory health research networks: a scoping review
Source: Syst Rev. 2022 Mar 6;11:40. doi: 10.1186/s13643-022-01910-x (PMC8900447; doi:10.1186/s13643-022-01910-x)
Supplement: Supplementary file 3 — Additional file 3. Individual study findings from the extracted literature. [file 13643_2022_1910_MOESM3_ESM.docx]

**Additional File 3** Individual study findings from the extracted literature, Social Network (SN) or Community-Based Participatory Research (CBPR), or Both (SN and CBPR)

| Case | Country | Empirical or  Non-Empirical | Aims/Purpose | Type of Stakeholder | SN or CBPR | Conceptualisation | Operationalisation | Measurement | Outcome |
| --- | --- | --- | --- | --- | --- | --- | --- | --- | --- |
| 1^(29)^ | China | Empirical | This research applies social network analysis (SNA) to characterize the Aging in Place (AIP) stakeholders in Nanjing, China, in order to help policymakers identify specific issues that exist in the AIP system. | Stakeholders from 23 categories and include service associations, government departments, information providers, older people. service providers, training agencies, assessment agencies, and social media. | SN | ST C1.1 Context specific > Within individuals ST C2.4 Relational > Reliability ST C2.5 Relational > Ability ST C2.6 Relational > Strength and quality of relationship ST C3.2 Complex concept > Multidimensional | ST O1.1 Context specific> Within individuals  ST O2.4 Relational > Reliability ST O2.6 Relational > Strength and quality of relationship | ST M1.1 Type of measure > Survey ST M1.2 Type of measure > Scaling ST M1.3 Type of measure > Qualitative ST M2.2 Level of measure > Ordinal | ST R2.2 Relational > Cohesion ST R4.1.3 Social Network Analysis > Individual level > Asymmetry ST R4.1.4 Social Network Analysis > Individual level > Centrality |
| 2^(31)^ | USA | Empirical | Two studies aimed at understanding consequences of giving students complete decision-making authority to select team members for a team assignment. | Undergraduate business students | SN | ST C1.1 Context specific > Within individuals ST C2.1 Relational > Trustworthy ST C2.2 Relational > Vulnerability ST C2.3 Relational > Integrity ST C2.4 Relational > Reliability ST C2.5 Relational > Ability ST C3.1 Complex concept > Multiplicities of trust | ST O2.2 Relational > Vulnerability ST O2.4 Relational > Reliability ST O2.5 Relational > Ability | ST M1.1 Type of measure > Survey ST M1.2 Type of measure > Scaling ST M2.2 Level of measure > Ordinal | ST R2.1 Relational >  Trustworthiness ST R2.5 Relational > Reliability |
| 3^(30)^ | Sweden | Empirical | To contribute to our understanding of flood risk governance by investigating the social organization of formal actors that contribute to mitigating urban flood risk in Swedish municipalities. | Individuals contributing to mitigating flood risk in Lomma Municipality as part of their professional activities or as significant property owners in the catchment area of the river running through Lomma town | SN | ST C2.2 Relational > Vulnerability ST C2.4 Relational > Reliability ST C3.2 Complex concept > Multidimensional | ST O2.4 Relational > Reliability | ST M1.2 Type of measure > Scaling ST M1.3 Type of measure > Qualitative ST M2.2 Level of measure > Ordinal | ST R2.3 Relational >  Relationship quality and type ST R2.4 Relational > Support ST R2.6 Relational > Ability |
| 4^(32)^ | China | Empirical | To understand how the closure-trust association covaries with individual respondent differences: How do differences in the network context for trust covary with respondent differences such that what is guanxi to one kind of person need not be guanxi to another? | Entrepreneurs operating  manufacturing firms | SN | ST C1.1 Context specific> Within individuals ST C3.2 Complex concept > Multidimensional | ST O2.6 Relational > Strength and quality of relationship | ST M1.1 Type of measure > Survey ST M1.2 Type of measure > Scaling ST M2.2 Level of measure > Ordinal | ST R4.3.1 Social Network Analysis >  Network level > Network size ST R4.3.3 Social Network Analysis > Network level > Closure ST R4.3.5 Social Network Analysis > Network level > Density |
| 5^(33)^ | USA | Empirical | To investigate the development of trust among residential environmental education (EE) program participants in two school groups, some of whom had initial familiarity with each other. | Students in the 6th grade from two schools | SN | ST C1.1 Context specific >Within individuals ST C1.2 Context specific > Surrounding individuals ST C2.2 Relational > Vulnerability ST C2.3 Relational > Integrity ST C2.7 Relational > Shared values, vision, and goals | ST O1.1 Context specific > Within individuals ST O2.2 Relational > Vulnerability ST O2.3 Relational > Integrity ST O2.4 Relational > Reliability ST O2.6 Relational > Strength and quality of relationship ST O4.1 Social Network Analysis > Reciprocal trust | ST M1.1 Type of measure > Survey ST M1.2 Type of measure > Scaling ST M1.3 Type of measure > Qualitative ST M1.4 Type of measure > Unobtrusive ST M2.2 Level of measure > Ordinal | ST R1.2 > Context specific >  Surrounding individuals ST R2.3 Relational >  Relationship quality and type ST R2.7 Relational > Integrity ST R4.1.2 Social Network Analysis > Individual level > Reciprocal trust |
| 6^(34)^ | Canada | Empirical | To examine if organisations who reside in the same sector and organisations who indicate higher levels of trust with other organisations are more likely to collaborate. | A swimming network that comprised 32 organisations involved in direct service provision | SN | ST C1.2 Context specific > Surrounding individuals ST C2.2 Relational > Vulnerability ST C2.3 Relational > Integrity ST C3.2 Complex concept > Multidimensional  ST C4.1 Social Network Analysis > Reciprocal trust | ST O1.2 Context specific > Surrounding individuals ST O2.6 Relational > Strength and quality of relationship | ST M.1.1 Type of measure > Survey ST M1.2 Type of measure > Scaling ST M2.2 Level of measure > Ordinal | ST R2.2 Relational > Cohesion ST R4.1.4 Social Network Analysis > Individual level > Centrality ST R4.2.2 Social Network Analysis > Group Level > Fragmentation ST R4.3.4 Social Network Analysis > Network level > Homophily (not supported) ST R4.3.6 Social Network Analysis > Network level > Centralisation |
| 7^(35)^ | Uganda | Empirical | The aims of this paper are two-fold. First, we describe in detail how to design and implement an evaluation of health partnerships, drawing on a specific theoretical framework (the ‘partnership framework’) and social network analysis (SNA). Second, we apply the partnership framework to a specific national level case of an immunization partnership to demonstrate how the framework can be used to describe and relate partnership domains to perceptions of outcomes in the process, and to assess the feasibility and usefulness of this approach. | Individuals involved in the HPV vaccine application process | BOTH: SN and CBPR | ST C2.3 Relational > Integrity ST C2.4 Relational > Reliability ST C2.5 Relational > Ability  ST C3.1 Complex concept > Multiplicities of trust | ST O1.1 Context specific > Within individuals ST O1.2 Context specific > Surrounding individuals ST O2.3 Relational > Integrity ST O2.4 Relational > Reliability ST O2.5 Relational > Ability ST O2.6 Relational > Strength and quality of relationship | ST M.1.1 Type of measure > Survey ST M1.2 Type of measure > Scaling ST M1.3 Type of measure > Qualitative ST M2.2 Level of measure > Ordinal | ST R2.8 Relational >  Shared values, visions and goals  ST R2.11 Relational > Power sharing and co-ownership |
| 8^(36)^ | USA | Empirical | The purpose of this paper is to identify characteristics of the network involved in implementing the community health improvement plan (CHIP) in one large community at the onset of a CHIP intervention effort. | Health Improvement Partnership of Maricopa County (HIPMC) partner organizations | BOTH: SN and CBPR | ST C2.4 Relational > Reliability ST C2.7 Relational > Shared values, vision, and goals | ST O2.4 Relational > Reliability ST O2.7 Relational > Shared vision, values, and goals | ST M.1.1 Type of measure > Survey ST M1.2 Type of measure > Scaling ST M2.2 Level of measure > Ordinal ST M2.4 Level of measure > Ratio | ST R4.3.5 Social Network Analysis >  Network level > Density ST R4.3.6 Social Network Analysis > Network level > Centralisation |
| 9^(37)^ | Canada | Empirical | To model the systems of knowledge and belief involved in the phenomenology of trust in social networks. | Senior managers of a large multidivisional, multiregional Canadian telecommunications firm | SN | ST C2.3 Relational > Integrity ST C2.4 Relational > Reliability ST C2.5 Relational > Ability ST C3.2 Complex concept > Multidimensional | ST O2.3 Relational > Integrity ST O2.4 Relational > Reliability ST O2.5 Relational > Ability | ST M.1.1 Type of measure > Survey ST M1.2 Type of measure > Scaling ST M2.2 Level of measure > Ordinal | ST R2.2 Relational > Cohesion ST R2.7 Relational > Integrity ST R4.1.1 Social Network Analysis > Individual level > Constraint ST R4.1.4 Social Network Analysis > Individual level > Centrality ST R4.3.5 Social Network Analysis > Network level > Density |
| 10^(38)^ | USA | Empirical | This article examines the impact that third-party relationships have on the development on interpersonal trust. | Social workers working for public and nonprofit human service agencies | SN | ST C2.1 Relational > Trustworthy ST C2.2 Relational > Vulnerability ST C2.5 Relational > Ability ST C3.2 Complex concept > Multidimensional | ST O1.1 Context specific > Within individuals ST O1.2 Context specific > Surrounding individuals ST O2.1 Relational > Trustworthiness ST O4.5 Social Network Analysis > Transferability | ST M.1.1 Type of measure > Survey ST M1.2 Type of measure > Scaling ST M2.1 Level of measure > Nominal | ST R1.1 Context specific > Within individuals ST R2.1 Relational > Trustworthiness ST R2.2 Relational > Cohesion ST R2.5 Relational > Reliability ST R4.1.5 Social Network Analysis > Individual level > Transferability |
| 11^(39)^ | USA | Empirical | This article presents a comparative analysis of the evolution of two community mental health networks that both have similar contracts from the State of Arizona. | Two community mental health networks that both have similar contracts from the State of Arizona and are governed by Network Administrative Organizations (NAOs) | SN | ST C1.1 Context specific >Within individuals ST C1.2 Context specific > Surrounding individuals ST C2.1 Relational > Trustworthy ST C2.2 Relational > Vulnerability | ST O2.1 Relational > Trustworthiness ST O2.6 Relational > Strength and quality of relationship | ST M.1.1 Type of measure > Survey ST M1.2 Type of measure > Scaling ST M1.3 Type of measure > Qualitative ST M2.2 Level of measure > Ordinal | ST R2.1 Relational > Trustworthiness ST R2.3 Relational >  Relationship quality and type |
| 12^(40)^ | Turkey | Empirical | To measure and analyse personal trust between co-workers (reciprocal trust) and its complementary risk by analysing subjective trust relations in an organisation. | A medium size enterprise in the textile sector | SN | ST C1.1 Context specific > Within individuals ST C1.2 Context specific > Surrounding individuals ST C2.2 Relational > Vulnerability ST C2.3 Relational > Integrity ST C2.6 Relational > Strength and quality of relationship ST C3.2 Complex concept > Multidimensional  ST C4.1 Social Network Analysis > Reciprocal trust ST C4.2 Social Network Analysis > Asymmetry | ST O1.2 Context specific > Surrounding individuals ST O2.3 Relational > Vulnerability ST O2.3 Relational > Integrity ST O2.4 Relational > Reliability ST O2.5 Relational > Ability ST O2.6 Relational > Strength and quality of relationship ST O2.6 Relational > Strength and quality of relationship ST O3.1 Complex concept > Multiplicities of trust ST O4.1 Social Network Analysis > Reciprocal trust | ST M.1.1 Type of measure > Survey ST M1.2 Type of measure > Scaling ST M2.2 Level of measure > Ordinal | ST R2.2 Relational > Cohesion ST R2.5 Relational > Reliability ST R2.7 Relational > Integrity ST R3.1 Complex concept > Multidirectional ST R4.1.2 Social Network Analysis > Individual level > Reciprocal trust ST R4.2.1 Social Network Analysis > Group level > Cliques |
| 13^(41)^ | USA | Empirical | To better understand the determinants of  interpersonal trust by explicitly recognizing that each dyad is embedded in a complex network of relationships with third parties. | A medium-sized, European-owned company with U.S. headquarters in the eastern United States | SN | ST C2.1 Relational > Trustworthy ST C2.3 Relational > Integrity ST C2.4 Relational > Reliability | ST O1.2 Context specific > Surrounding individuals ST O2.1 Relational > Trustworthiness ST O2.3 Relational > Integrity ST O2.4 Relational > Reliability ST O4.3 Social Network Analysis > Structural Evidence ST O4.4 Social Network Analysis > Network Closure ST O4.5 Social Network Analysis > Transferability | ST M.1.1 Type of measure > Survey ST M1.2 Type of measure > Scaling ST M2.2 Level of measure > Ordinal | ST R.4.1.5 Social Network Analysis > Individual level > Transferability ST R4.2.3 Social Network Analysis > Group Level > Structural Equivalence ST R4.2.4 Social Network Analysis > Third party relationships |
| 14^(42)^ | Canada | Non-empirical | To propose a theoretical model that can structure the evaluation of the processes and outcomes of organizational participatory research (OPR) health partnerships. | N/A | CBPR | ST C1.2 Context specific > Surrounding individuals ST C2.7 Relational > Shared values, vision, and goals ST C2.8 Relational > Power sharing and co-ownership | ST O1.2 Context specific > Surrounding individuals ST O2.7 Relational > Shared vision, values, and goals ST O2.8 Relational > Power sharing + co-ownership | ST M.1.1 Type of measure > Survey ST M1.2 Type of measure > Scaling ST M1.3 Type of measure > Qualitative ST M2.1 Level of measure > Nominal ST M2.2 Level of measure > Ordinal ST M2.3 Level of measure > Open-ended question | ST R1.2 > Context specific >  Surrounding individuals ST R2.8 Relational >  Shared values, visions and goals  ST R2.10 Relational > Power sharing and co-ownership ST R2.11 Relational > Sustainability |
| 15^(43)^ | USA | Empirical | Primary aim was to include the perspectives of major types of stakeholders in order to identify factors that contribute to trust within community-academic research partnerships from the perspectives of community members’, academic researchers’ and healthcare providers’.  Secondary aim was to evaluate the relative importance of the identified factors for creating and maintaining trust within partnerships as well as for improving public trust in research more generally. | This study involved five NIH-funded Clinical and Translational Science Award (CTSA) grantees | CBPR | ST C1.1 Context specific > Within individuals ST C1.2 Context specific > Surrounding individuals ST C2.2 Relational > Vulnerability ST C2.3 Relational > Integrity ST C3.2 Complex concept > Multidimensional | ST O1.1 Context specific > Within individuals ST O2.6 Relational > Strength and quality of relationship ST O2.8 Relational > Power sharing + co-ownership ST O4.1 Social Network Analysis > Reciprocal trust | ST M1.2 Type of measure > Scaling ST M1.3 Type of measure > Qualitative ST M2.2 Level of measure > Ordinal ST M2.3 Level of measure > Open-ended question | ST R2.2 Relational > Cohesion ST R2.8 Relational >  Shared values, visions and goals  ST R2.9 Relational > Problem solving ST 2.11 Relational > Sustainability ST R4.1.2 Social Network Analysis > Individual level > Reciprocal trust |
| 16^(44)^ | USA | Empirical | In this article, we have described our mixed methods study of CBPR processes and outcomes, with the aim of testing the CBPR conceptual model and the variability of research partnerships across the United States. | Principal investigators of CBPR partnerships and case studies | CBPR | ST C1.2 Context specific > Surrounding individuals ST C2.7 Relational > Shared values, vision, and goals ST C2.8 Relational > Power sharing + co-ownership ST C3.1 Complex concept > Multiplicities of trust  ST C3.2 Complex concept > Multidimensional | ST O1.2 Context specific > Surrounding individuals ST O2.4 Relational > Reliability ST O2.5 Relational > Ability ST O2.6 Relational > Strength and quality of relationship ST O3.1 Complex concept > Multiplicities of trust | ST M1.1 Type of measure > Survey ST M1.2 Type of measure > Scaling ST M1.3 Type of measure > Qualitative ST M2.1 Level of measure > Nominal | ST R2.10 Relational > Power sharing and co-ownership ST R3.1 Complex concept > Multidirectional |
| 17^(45)^ | USA | Empirical | The purpose of this case study is to describe the first-year planning process, to explore member experiences and determine key facilitators and barriers during the Partnering for Obesity Planning and Sustainability Community Advisory Board's (POPS-CAB’s) early development, and to inform CBPR best practices for capacity building. | Six local organizations, identified for their missions related to child health and services that target low socioeconomic families | CBPR | ST C1.2 Context specific > Surrounding individuals ST C2.3 Relational > Integrity ST C2.4 Relational > Reliability ST C2.7 Relational > Shared values, vision, and goals | ST O1.1 Context specific > Within individuals ST O1.2 Context specific > Surrounding individuals ST O2.3 Relational > Integrity ST O4.1 Social Network Analysis > Reciprocal trust | ST M1.1 Type of measure > Survey ST M1.3 Type of measure > Qualitative ST M1.2 Type of measure > Scaling ST M2.2 Level of measure > Ordinal | ST R2.2 Relational > Cohesion ST R2.6 Relational > Ability ST R2.8 Relational >  Shared values, visions and goals  ST R2.9 Relational > Problem solving ST R2.11 Relational > Sustainability |
| 18^(46)^ | North America | Empirical | 1a) To characterize trustworthiness within community-academic research partnerships, and b) to identify institutional barriers to trustworthiness encountered by such partnerships, and approaches taken to overcome those barriers; 2) To develop a measure of researcher trustworthiness in community-academic research partnerships; and 3) To identify opportunities to cultivate trustworthiness within a case of genetic research, APOL1 testing for End-Stage Renal Disease risk in African American communities. | AIM 1:  Individuals who have experience as a community partner, academic partner, or who identified with both community and academic roles (termed “bridge” partners, based on one participant’s self-identification,) within research partnerships  AIM 2:  Diverse community members in Seattle | CBPR | ST C1.1 Context specific > Within individuals ST C1.2 Context specific > Surrounding individuals ST C2.1 Relational > Trustworthy ST C2.2 Relational > Vulnerability ST C2.8 Relational > Power sharing and co-ownership ST C3.2 Complex concept > Multidimensional | ST O1.1 Context specific > Within individuals ST O1.2 Context specific > Surrounding individuals ST O2.1 Relational > Trustworthiness | ST M1.1 Type of measure > Survey ST M1.2 Type of measure > Scaling ST M1.3 Type of measure > Qualitative ST M2.2 Level of measure > Ordinal | ST R2.1 Relational >  Trustworthiness ST R2.5 Relational > Reliability ST R2.10 Relational > Power sharing and co-ownership ST R2.12 Relational > Vulnerability |
| 19^(54)^ | USA | Empirical | The purpose of this study was to advance the understanding of trust in CBPR partnerships by exploring the relationship between trust and communication ethics. | FOR CASE STUDY (Interviews): People who were actively involved in the CBPR project as a faculty/staff member, volunteer, or advisory board member for individual interviews. FOR SURVEY: The final database consisted of principal investigators and community or academic contacts | CBPR | ST C1.1 Context specific > Within individuals ST C1.2 Context specific > Surrounding individuals ST C2.2 Relational > Vulnerability ST C2.3 Relational > Integrity ST C2.7 Relational > Shared values, vision, and goals ST C3.1 Complex concept > Multiplicities of trust ST C3.2 Complex concept > Multidimensional | ST O1.1 Context specific > Within individuals ST O1.2 Context specific > Surrounding individuals ST O2.3 Relational > Integrity ST O2.4 Relational > Reliability ST O2.5 Relational > Ability ST O3.1 Complex concept > Multiplicities of trust | ST M1.1 Type of measure > Survey ST M1.3 Type of measure > Qualitative ST M1.2 Type of measure > Scaling ST M2.1 Level of measure > Nominal ST M2.2 Level of measure > Ordinal | ST R2.2 Relational > Cohesion ST R2.8 Relational >  Shared values, visions and goals  ST R2.10 Relational > Power sharing and co-ownership ST R3.1 Complex concept > Multidirectional |
| 20^(47)^ | Malawi | Empirical | The purpose of this research was to explore perspectives of  NGO staff in order to understand barriers and facilitators in their work with communities. | Participant observations  and interviews were conducted with NGO1 staff, volunteers and partners in the US and Malawi | CBPR | ST C2.3 Relational > Integrity ST C2.4 Relational > Reliability ST C2.5 Relational > Ability ST C2.7 Relational > Shared values, vision, and goals | ST O1.1 Context specific > Within individuals | ST M1.3 Type of measure > Qualitative ST M1.4 Type of measure > Unobtrusive ST M2.3 Level of measure > Open-ended question | ST R2.2 Relational > Cohesion ST R2.3 Relational > Relationship quality and type ST R2.5 Relational > Reliability ST R2.7 Relational > Integrity ST R2.8 Relational > Shared values, visions and goals  ST R2.10 Relational > Power sharing and co-ownership ST R2.11 Relational > Sustainability |
| 21^(48)^ | USA | Empirical | This study was designed to investigate relationships between dimensions of facilitation management (logistical arrangements, social support, participatory discussion, conflict resolution, and participatory decision-making) and interorganizational coordination and trust, among the members of the Twin Cities anti-Iraq War non-profit network. | Iraq War movement members in the Twin Cities | SN | ST C1.2 Context specific > Surrounding individuals ST C2.3 Relational > Integrity ST C3.1 Complex concept > Multiplicities of trust | ST O1.1 Context specific > Within individuals ST O1.2 Context specific > Surrounding individuals ST O2.4 Relational > Reliability ST O4.1 Social Network Analysis > Reciprocal trust | ST M1.1 Type of measure > Survey ST M1.2 Type of measure > Scaling ST M2.2 Level of measure > Ordinal ST M2.3 Level of measure > Open-ended question | ST R2.2 Relational > Cohesion ST R2.4 Relational > Support ST R2.5 Relational > Reliability ST R2.8 Relational > Shared values, visions and goals  ST R2.9 Relational > Problem solving ST R2.10 Relational > Power sharing and co-ownership ST R4.3.5 Social Network Analysis > Network level > Density |
| 22^(49)^ | Australia | Empirical | The current study addresses and extends the “need to think of mechanisms and processes which can reinforce and help sustain team-based initiatives” (McHugh, Niehaus, & Swiercz, 1997, p. 47) but also thinking through those mechanisms that undermine them, and articulates such mechanisms and processes theoretically in terms of structures of intra-team trust relations. | Three Australian Football League (AFL) teams from Melbourne, Australia | SN | ST C2.7 Relational > Shared values, vision, and goals ST C3.1 Complex concept > Multidimensional | ST O1.2 Context specific > Surrounding individuals ST O4.1 Social Network Analysis > Reciprocal trust | ST M1.1 Type of measure > Survey ST M1.2 Type of measure > Scaling ST M2.1 Level of measure > Nominal | ST R2.5 Relational > Reliability ST R4.1.2 Social Network Analysis > Individual level > Reciprocal trust ST R4.1.4 Social Network Analysis > Individual level > Centrality ST R4.3.3 Social Network Analysis > Network level > Closure ST R4.3.4 Social Network Analysis > Network level > Homophily |
| 23^(50)^ | USA and China | Empirical | We problematize in this study a long-held assumption of the close affinity between social capital and trust.  Through a systemic empirical test, we examine if they may be decoupled. | Adults ages 21-64 living in China or USA | SN | ST C1.1 Context specific > Within individuals  ST C1.2 Context specific > Surrounding individuals ST C2.1 Relational > Trustworthy ST C3.1 Complex concept > Multiplicities of trust | ST O1.1 Context specific > Within individuals ST O1.2 Context specific > Surrounding individuals ST O3.1 Complex concept > Multiplicities of trust | ST M1.1 Type of measure > Survey ST M1.2 Type of measure > Scaling ST M1.3 Type of measure > Qualitative ST M2.2 Level of measure > Ordinal | ST R1.2 > Context specific >  Surrounding individuals ST R4.3.3 Social Network Analysis > Network level > Closure |
| 24^(51)^ | Germany | Empirical | To show how a combination of quantitative and qualitative methods of social network analysis can collect data on trust in networks. | Five municipalities that have been awarded by a jury as part of the United Nations Decade of Education for Sustainable Development (UNDESD) | SN | ST C1.2 Context specific > Surrounding individuals ST C2.3 Relational > Integrity ST C2.6 Relational > Strength and quality of relationship ST C2.7 Relational > Shared values, vision, and goals ST C3.1 Complex concept > Multiplicities of trust ST C3.2 Complex concept > Multidimensional | ST O1.1 Context specific > Within individuals ST O1.2 Context specific > Surrounding individuals | ST M1.1 Type of measure > Survey ST M1.2 Type of measure > Scaling ST M1.3 Type of measure > Qualitative ST M2.1 Level of measure > Nominal | ST R2.2 Relational > Cohesion ST R2.5 Relational > Reliability ST R2.8 Relational > Shared values, visions and goals  ST R4.3.1 Social Network Analysis >  Network level > Network size ST R4.3.2 Social Network Analysis >  Network level > Structural holes |
| 25^(52)^ | Ge Germany | Empirical | To compare several acclaimed, though to a certain degree competing, theoretical insights in interpersonal intra-organizational trust dynamics. More specifically, they aimed to model the evolution of trust relationships, i.e. relational choices between two actors. | Members of the management team of a paper factory | SN | ST C1.1 - Context specific > Within individuals  ST C1.2 Context specific > Surrounding individuals ST C2.7 Relational > Shared values, vision, and goals ST C3.1 Complex concept > Multiplicities of trust ST C4.1 Social Network Analysis > Reciprocal trust | ST O1.2 Context specific > Surrounding individuals ST O2.3 Relational > Integrity ST O2.4 Relational > Reliability ST O2.6 Relational > Strength and quality of relationship ST O4.2 Social Network Analysis > Homophily | ST M1.1 Type of measure > Survey ST M1.2 Type of measure > Scaling ST M2.2 Level of measure > Ordinal | ST R4.1.1 Social Network Analysis >  Individual level > Constraint ST R4.1.2 Social Network Analysis > Individual level > Reciprocal trust  ST R4.1.3 Social Network Analysis > Individual level > Asymmetry  ST R42.1 Social Network Analysis > Group Level > Cliques ST R43.2 Social Network Analysis > Network level > Structural holes |
| 26^(53)^ | N/A | Non-empirical | Trust networks and some basic techniques for measuring them. We will discuss methods for processing network data and identify some common types of network analyses and measures. | N/A | SN | ST O1.2 Context specific > Surrounding individuals ST C2.2 Relational > Vulnerability ST C2.6 Relational > Strength and quality of relationship ST C3.1 Complex concept > Multiplicities of trust | ST O2.6 Relational > Strength and quality of relationship | ST M1.1 Type of measure > Survey ST M1.2 Type of measure > Scaling ST M2.2 Level of measure > Ordinal | N/R |

*Legend:* SN: Social Network, SNA: Social Network Analysis, CBPR: Community-based Participatory Research, N/R: Not Reported, ST: Sub-Theme, C(#): Conceptualisation of Trust, O(#): Operationalisation of Trust, M(#): Measurement of Trust, R(#): Outcome pertaining to Trust
